# Electrostatic adhesion mitigates aerodynamic losses from gap formations in feathered wings

**DOI:** 10.1038/s44172-025-00452-z

**Published:** 2025-10-13

**Authors:** Kevin P. T. Haughn, Jeffrey T. Auletta, John T. Hrynuk, Todd C. Henry

**Affiliations:** 1 DEVCOM Army Research Lab, Aberdeen Proving Ground, Aberdeen, MD USA; 2 Department of Mechanical Engineering, Rose-Hulman Institute of Technology, Terre Haute, IN, USA

**Keywords:** Aerospace engineering, Physiology

## Abstract

Birds morph the shape of their wings during flight to achieve impressive maneuverability and adapt to dynamic environments, such as cities and forests. Engineers have explored using avian-inspired designs with feather-based wing morphing to achieve similar capabilities with small uncrewed aircraft. However, engineered feather designs haven’t incorporated the microscopic structural features that prevent feather separation for natural fliers within dynamic airflows and during wing shape changes. Without a fastening mechanism, gaps can form throughout the wing’s surface that impair maneuverability and shorten flight range. Here we show how electrostatic feather fastening adapts aerodynamic force generation to improve maneuverability and efficiency. Further, the electrostatically adhered feathers offered a preferable relationship with velocity, improving on passive feather aerodynamics and often generating responses comparable or favorable to the baseline engineered wing at higher flow speeds. As small aircraft are expected to fly faster, further, and with advanced aerobatic capability, feathered morphing wings incorporating electrostatic adhesion will advance aircraft adaptability for successful operation in complex environments.

## Abstract (web summary)

Bird wings use microscopic hooks to maintain connection between feathers during flight. Kevin PT Haughn and colleagues report an electro-adhesive alternative for engineered morphing wings, thus fastening feathers to improve manoeuvrability in flight.

## Introduction

Complex and dense environments, such as forests and cities, remain challenging for small fixed-wing aircraft due to the large number of obstacles and gusts of wind^1–5^. Currently, incorporating small uncrewed aerial systems (UAS) in these challenging locations would fall on multi-copter designs because of their greater agility. However, multi-copter flight relies entirely on the vertical thrust provided by several rotors, offering a power-hungry means to achieve flight that limits range and endurance^6^. For objectives that require a greater range, fixed-wing designs provide superior performance, using large surfaces to generate a vertical lift force from forward velocity during flight. This requires less power to stay aloft, increasing range and endurance. However, these designs are traditionally less maneuverable, defined here as the degree to which a UAS may change its velocity vector^7^. For this reason, engineers have returned their focus to drawing inspiration from our predecessors in flight, birds.

**Fig. 1:**
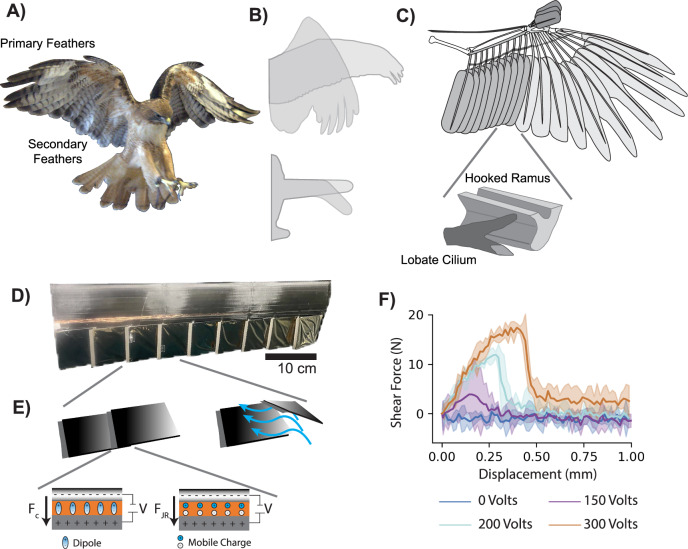
Electrostatic adhesion fastens engineered feathers together for avian-informed wing design. **A** Bird wings incorporate a series of overlapping feathers that slide over one another to maintain contact and produce a smooth, unified lifting surface during flight (Red-tailed hawk at the California Raptor Center Credit: Alfonso Martínez, BIRD Lab, UC Davis). **B** The ability to exhibit large shape changes to their primary lifting surfaces has inspired small uncrewed aerial system designs to incorporate feathered structures to achieve similar degrees of freedom during flight. **C** On a bird’s wing, this effect is achieved by incorporating both a ligament structure connecting the hollow shaft at the base of the feathers, as well as hook and barbule structures that act as directional probabilistic fasteners (figure adapted from refs. ^16,25^). **D** We used electrostatic adhesion to achieve a similar effect for an engineered wing with a feathered trailing edge. **E** This system used a low power voltage (~0.72 mW) to create two adhesive forces, including coulombic (${F}_{{\rm {C}}}$) and Johnsen–Rahbek (${F}_{{{\rm {JR}}}}$), allowing the wing to toggle between a continuous trailing edge structure when activated, and low traction movement when inactive. **F** The bond between feathers increased with voltage, requiring greater force to create separation. Shaded regions represent 95% confidence intervals (1.96$\,\times$ standard deviation, *N* = 5).

Birds can rapidly change direction to avoid trees in forests while hunting and fly through gusts of wind with minimal trajectory perturbation^7–11^. Relatedly, birds modulate their stability in flight by changing wing shape to achieve maneuverability and resilience^8,12–14^ (Fig. 1A). This capability has inspired the field of morphing UAS to develop maneuverable, resilient, and adaptive aircraft through controlled shape change^15–17^ (Fig. 1B). Active materials and smart structures have played a large role in shaping the field of morphing aircraft to achieve smooth shape changes for improved efficiency and gust rejection^17–23^. These methods work well for small shape changes, such as chordwise wing curvature (camber) and thickness morphing. However, material properties are challenged as shape changes grow larger, requiring both compliance and stiffness to achieve a new shape while simultaneously resisting deformation from external aerodynamic forces^22,24^. Recently, there has been a push toward greater biological inspiration in UAS design, flying at lower Reynolds numbers (${\mathrm{Re}}$) and using overlapping feathers, or feather-like plates, to achieve relatively large-scale shape changes without sacrificing structural integrity^25–31^. These designs no longer use the aileron deflectors traditionally found on aircraft wings for roll control. Instead, the wing extension and sweep changes offered by the overlapping feather designs are used to generate roll and pitching moments. These large-scale shape changes have shown that feather-inspired morphing can improve maneuverability in aerobatic UAS flight^31–33^. However, without a proper fastening mechanism, gaps can form between the feathers, thus disrupting the structure and aerodynamics of the aircraft’s primary lifting surface.

Streamwise gap formation on a rigid wing can reduce lift as the angle of attack, *α*, increases^34–36^. Similarly, disconnecting thin feather-like structures reduces their combined stiffness, which can result in a more compliant trailing edge and ultimately reduce lift at higher flow speeds^37^. Although these effects can benefit gust alleviation, the result limits an aircraft’s ability to rapidly change direction. Therefore, optimal performance during aerobatic flight requires the feather connection to be maintained. Natural feathers incorporate two interacting structures combining the lobate cilium of a distal barbule and hooked ramus to create a one-dimensional fastening effect, allowing the feathers to slide over one another without separating^25,36,38,39^ (Fig. 1C). For this reason, some morphing UAS have natural feathers incorporated into their design^26,30^. Still, an active means for inter-feather fastening has yet to be explored and incorporated on synthetic feathered wings.

**Fig. 2:**
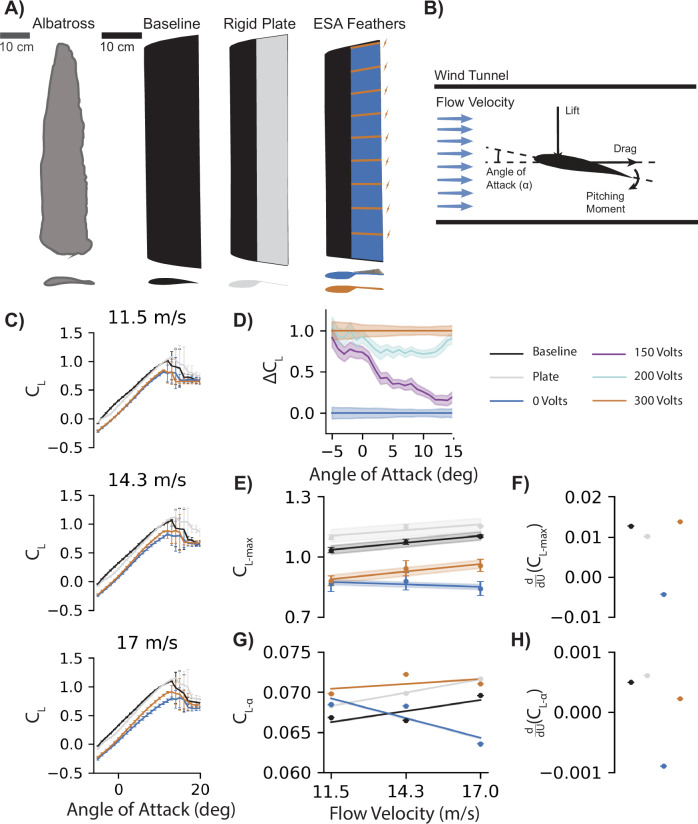
Active electrostatic adhesion (EA) improved feathered wing lift generation at higher speeds. **A** We used an avian-inspired baseline airfoil shape (Wortmann FX 60-126: black) to construct three half-span wings. A rigid plate design (gray) provided an inflexible feather-like airfoil geometry. An adaptive feather design enabled passive feather separation (blue) and EA feather fastening (orange)^43^. **B** Force and moment measurements were taken at three flow velocities (11.5, 14.3, 17 m/s) and angles of attack from −5° to 20°. **C** Increasing flow speed illuminated differences between passive and active EA feathered wings (*N* ~ 31,000). **D** Reducing EA voltage (200 V: cyan, 150 V: purple) reduced the normalized change in lift (Δ${C}_{{\rm {L}}}$) between passive (0 V) and EA fastened feathers at 17 m/s. Shaded regions and error bars represent 95% confidence intervals. **E** The maximum lift coefficient (${C}_{{\rm {L}}-\max }$) increased for the active EA wing but decreased for the passive wing. **F** ${C}_{{\rm {L}}-\max }$ increased most rapidly for the active EA wing, reversing the negative relationship between ${C}_{{\rm {L}}-\max }$ and velocity for the feathered wing. **G** The relationship between lift and angle of attack (${C}_{{\rm {L}}-\alpha }$) was strongest for the feathered wings at 11.5 m/s but weakened with increased velocity. **H** Active EA effectively eliminated the negative relationship between ${C}_{{\rm {L}}-\alpha }$ and velocity.

Here, we used electrostatic adhesion (EA) as a low power (~0.72 mW) means to modulate the connection between feathers on the trailing edge of a wing (Fig. 1D). EA results from the application of an electric potential across two conducting electrode surfaces when separated by a dielectric material (Fig. 1E). EA has been used in robotics and other aerial vehicle applications for clutching flat plates, locking them in place when EA is applied^40,41^. Increasing the voltage applied to the EA feathers produced greater peak forces prior to separation (Fig. 1F). We investigated how EA (0, 150, 200, 300 V) offers an engineered analog to biological feather fastening and can improve the aerodynamic performance of a feathered wing during flight. However, birds are not optimized to fly based on flight metrics derived through extensive design consideration^42^. Therefore, the feathered wing differs notably from traditionally engineered wings in both airfoil geometry and structural compliance under aerodynamic loading. For this reason, we cannot expect the feathered wings to outperform an engineered design in traditional aerodynamic metrics. Instead, we have included two wings to act as baselines from which we can contrast the effects of EA on the feathered wing’s performance. Of the two rigid baseline wings, one shares the airfoil geometry of the feathered wing and the other was built with an established avian-inspired airfoil shape, Wortman FX 60-126 (Fig. 2A)^26,43^. The various wings used for comparison allowed us to differentiate between aerodynamic effects caused by feather gap formation, structural compliance^37^, and airfoil geometry. Aerodynamic force measurements were collected on each half-span wing model in a wind tunnel at angles of attack ranging from −5° to 20°, and at three flow velocities (11.5, 14.3, and 17 m/s) ensuring comparable aerodynamic scale to the current state of the art morphing aircraft^30,31^ (${10}^{5}\lesssim {\mathrm{Re}}\lesssim 1.5\times {10}^{5}$) (Fig. 2B). The aerodynamic effects caused by gap formation grew with flow velocity, resulting in increasingly improved maneuverability when using EA fastened feathers, instead of passive feathers without EA. Additionally, the EA's feathered wing improved efficiency as flow velocity increased. Therefore, incorporating EA into morphing UAS design can reduce the aerodynamic losses that occur from passive feather gap formation, ultimately expanding UAS adaptability to broader mission scopes and environments, such as dense forests and cities, where maneuverability is crucial for safe and effective operation.

## Results

### Aerodynamic characteristics vary with velocity

Aerodynamic effects scale with the squared flow velocity. We highlighted the unaccounted-for variations in performance as flow velocity increased by using aerodynamic coefficients. Comparing lift, ${\rm {L}}$, generation between the tested wings, we used the coefficient of lift

1 $${C}_{{\rm {L}}}=\frac{2L}{\rho {{SU}}^{2}},$$

normalized by the flow velocity, $U$, wing surface area, $S$, and air density, $\rho$. As expected, both the baseline and rigid plate wing produced significantly greater ${C}_{{\rm {L}}}$ than the feathered wing (Fig. 2C). The activated EA wing, operating at 300 V, produced similar ${C}_{{\rm {L}}}$ to the passive EA wing at the slowest flow velocity (11.5 m/s). This was likely due to the inherent feather stiffness preventing the feathers from bending and separating during flight. As the flow accelerated, a greater aerodynamic force led the passive feathers to bend and separate.

The voltage-driven nature of EA enables a degree of tunability for responding to mission needs or changes in the environment. For instance, using a voltage of 200 V at 17 m/s flow speed reduced the ${\varDelta C}_{{\rm {L}}}$ by ~25% between 2° and 13° angle of attack before returning to 90% of the 300 V ${\varDelta C}_{{\rm {L}}}$. Supplying 150 V caused individual feathers to separate gradually as the angle of attack increased. This resulted in a gradual reduction in ${C}_{{\rm {L}}}$, initially performing like the fully active EA wing until dropping to values within 16% of the fully passive 0 V feathered wing (Fig. 2D). This could offer an additional means for adaptive wing design through actively tuning the EA to produce various aerodynamic relationships between the fully active and passive bounds. However, the remainder of this work aims to determine the aerodynamic effects at the bounds of this relationship. Specifically, we aim to investigate the maximum performance boosts available for a feathered wing incorporating EA fastening at 300 V instead of passive feathers at 0 V.

Aircraft balance weight, lift, drag, and thrust to achieve an equilibrium state, known as trim, for steady flight at a desired cruise condition. This informs the general design for an aircraft, but it is necessary to consider how these aerodynamic forces change with flight conditions, such as during aerobatic maneuvers and gusts of wind. Thus, we considered the maximum lift generated, ${C}_{{\rm {L}}-\max }$, and each wing’s relationship between *C*_L_ and angle of attack, ${C}_{{\rm {L}}-\alpha }$, at various flow velocities (11.5, 14.3, and 17 m/s). We used linear mixed effects models in Python to compare these metrics between each wing with 95% confidence intervals (*N* ~ 31,000 per wing-velocity combination; *N* ~ 374,000 total). As expected, the rigid plate wing and the baseline wing produced the highest ${C}_{{\rm {L}}-\max }$ values, which grew with increasing velocity. The feathered wing generated reduced ${C}_{{\rm {L}}-\max }$ values whether the EA was activated or not (~0.87) at 11.5 m/s (Fig. 2E). The passive feathered wing reduced ${C}_{\rm {{L}}-\max }$ values further as flow velocity increased to 17 m/s, achieving only 76% of the baseline maximum lift. However, activating EA to enforce a constant connection between the feathers improved the feathered wing ${C}_{{\rm {L}}-\max }$ by 14%, reducing the ${C}_{{\rm {L}}-\max }$ loss by 46% when compared to the passive feathered wing’s loss from baseline. Further investigating the relationship between ${C}_{{\rm {L}}-\max }$ and velocity showed that the passive feathered wing held a negative relationship between maximum ${C}_{{\rm {L}}}$ and flow velocity. However, EA increased ${C}_{{\rm {L}}-\max }$ of the feathered wing at a significantly more rapid rate than even the rigid wings (*P* < 0.001), 8% faster than the baseline wing and 37% faster than the rigid plate wing (Fig. 2F).

Increasing velocity also changed the general relationship between ${C}_{L}$ and the angle of attack, ${C}_{L-\alpha }$, where the EA feathered wing produced the highest ${C}_{{\rm {L}}-\alpha }$ (Fig. 2G). Although the passive feathered wing held a similar α-sensitivity at the slowest flow velocity, its ${C}_{{\rm {L}}-\alpha }$ rapidly decreased as velocity increased. Activating EA fastening completely reversed the rate of α-sensitivity drop, leading the wing to behave more similarly to the rigid wings, showing a small increase in α-sensitivity with greater velocity (Fig. 2H). By each metric, the passive feathered wing experienced a decreasing relationship for the lift coefficient as flow velocity increased. However, activating EA for feather connection significantly improved the wing’s lift-generating capability as flow velocity increased. By preventing gaps from forming in the wing’s surface, the EA fastened wing behaved more like a stiffer continuous surface wing to achieve ${C}_{\rm {{L}}}$ relationships more comparable to the baseline and rigid plate wings^37^.

### Compliance and geometry affect efficiency

**Fig. 3:**
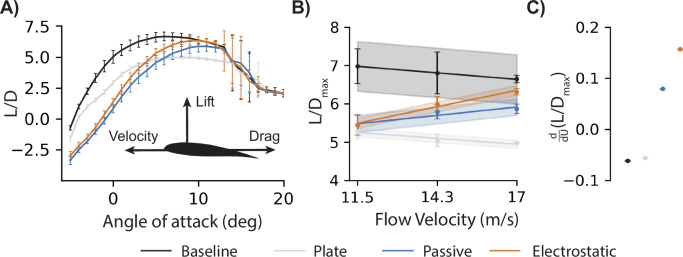
Active electrostatic adhesion (EA) improved efficiency at higher speeds. **A** The lift-to-drag ratio (*L*/*D*) measures the efficiency of a wing (*N* ~ 31,000) by comparing orthogonal aerodynamic forces responsible for balancing the weight and thrust requirements of an aircraft. **B** At low flow velocities (11.5 m/s), the baseline wing (black) was more efficient than the feathered wings (passive: blue; EA: orange); however, the rigid wings (baseline: black; plate: gray) lost efficiency as flow velocity increased, whereas the more compliant feathered wings became more efficient. **C** Electrostatic adhesion improved feathered wing efficiency at nearly twice the rate of the passive feathers (198%).

Although measuring lift provides crucial information regarding the performance of an aircraft wing, specifically regarding the maximum mass to be carried during flight and how rapidly the aircraft can accelerate orthogonally to the flight path, efficiency is another crucial metric to consider during aircraft design. Aircraft efficiency is often described by comparing the transverse lifting force, *L*, to the drag force, *D*, acting in the streamwise direction opposite to the aircraft’s velocity (Fig. 3A). The ratio between these two orthogonal forces (*L*/*D*) relates the aircraft mass capacity to the thrust required to maintain velocity. This intuitively provides a metric to gauge aircraft endurance, range, and power requirements. As expected, the baseline engineered wing consistently produced the greatest maximum *L*/*D* compared to the other wings (Fig. 3B). Notably, the rigid plate wing consistently achieved the lowest maximum *L*/*D* due to the high drag produced by the feather-like airfoil geometry when rigid (Fig. S1). The feathered wing produced a similar maximum *L*/*D* to the rigid plate wing at the lowest flow velocity when aerodynamic loading produced the least trailing edge deflection (difference within 4% and statistically insignificant according to a 95% confidence interval). As velocity increased, the two rigid wings grew less efficient, but the two feathered wings grew more efficient as the compliant trailing edge deflected under the aerodynamic loads, resulting in less drag than the two rigid wings (Fig. S1). Further, using EA to fasten the feathers produced only a small change in drag as velocity increased (${\varDelta C}_{{\rm {D}}}=0.01$ at 17 m/s), allowing the greater ${C}_{{\rm {L}}}$ offered by EA to improve the feathered wing’s efficiency at nearly double ($1.97$ times) the rate of the passive feathers (Fig. 3C). At 17 m/s, EA produced 17% greater *L*/*D* to achieve 95% of the maximum *L*/*D* for the engineered baseline (Fig. 3B). Therefore, feathered wing compliance was preferable over rigidity for the feather-like airfoil geometry in high-speed flight; however, this geometry did not achieve maximum efficiency until compliance and gap prevention were both achieved by incorporating EA into the feathered wing.

### Electrostatic feather fastening improves maneuverability

**Fig. 4:**
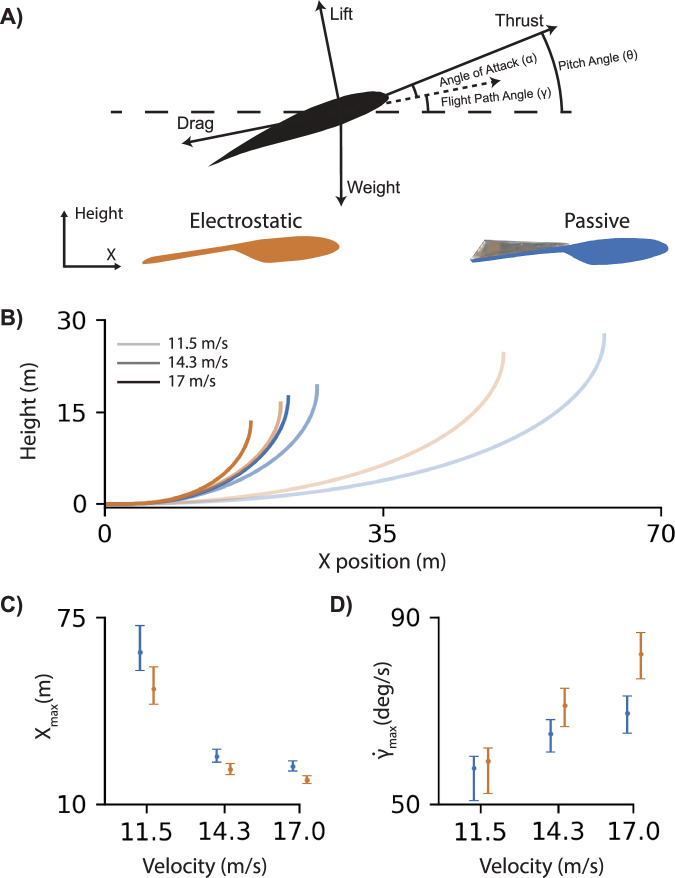
Electrostatic adhesion improved maneuverability of a feathered wing at higher speeds. **A** We used linear regression to model the lift and drag produced by the electrostatic (orange) and passive (blue) feathered wings. **B** Electrostatic adhesion (EA) enabled a smaller flight path radius during a pull-up maneuver at various flight speeds (11.5, 14.3, 17 m/s; distinguished by increased opacity). **C** The maximum *x*-distance (*X*_max_) traveled during the pull-up maneuver decreased with flight speed. This was because the overall change in angle of attack was limited by the difference between the maximum angle of attack (10°) and the trim angle of attack for each flight speed. In each case, EA feather fastening produced a smaller *X*_max_, with no overlap between respective 95% confidence intervals at the highest speed, as defined by the linear regression models. **D** The flight path angle change rate ($\dot{\gamma }$) increased with flight speed for both wings, but grew more rapidly for the EA feathered wing, producing a greater improvement in maneuverability at higher velocities (determined by 95% confidence interval).

Enforcing an electrostatic connection between feathers generated significant aerodynamic improvements for the feathered wing that can contribute to greater maneuverability in flight. To illustrate this effect, we constructed two longitudinal point-mass dynamics flight models to simulate rapid pull-up flight trajectories for the feathered wing with and without EA. These models assumed the wing was the primary source of lift and drag, neglecting the aerodynamic characteristics of a fuselage and tail. We used linear regression to model the ${C}_{{\rm {L}}}$ and ${C}_{{\rm {D}}}$ measurements and predict the lift and drag generated throughout the linear flight regime (−5° ≤ *α* ≤ 12°; 11.5 m/s ≤ $U$ ≤ 17 m/s) (Fig. S2). Additionally, the model assumed effective pitch control was achieved by an external control surface, such as an elevator located on the aircraft’s tail. Each flight model was initialized to begin in steady level flight for each considered flight velocity (11.5, 14.3, and 17 m/s) by calculating the thrust and angle of attack to balance the lift, drag, and mass (0.7 kg) for a flight path angle, $\gamma$, of zero degrees (Fig. 4A). Metric comparisons were determined significant using 95% confidence intervals provided by the linear regression models.

Here, we followed the definition of maneuverability as a metric measuring an aircraft’s ability to change its velocity vector^7^. Since streamwise acceleration is predominantly controlled by an aircraft’s thrust capability, we focused instead on a 90° change in flight path direction, throttling thrust to maintain speed through the rapid pull-up maneuver (Fig. 4B). We used PID feedback control to effectively adjust aircraft pitch rate so that angles of attack would consistently approach and then maintain an incidence of 10° during the pull up maneuver. This allowed the simulated aircraft to achieve high lift while remaining within the linear flight regime, thus preventing the occurrence of stall and maintaining model accuracy. We measured the effectiveness of both wings during this maneuver using spatial and temporal metrics, including the horizontal distance required to complete the maneuver, *X*_max_, and the rate of change in flight path angle, $\dot{\gamma }$_max_. According to these metrics, the improved maneuverability offered by EA grew significantly as the velocity increased, according to the 95% confidence intervals. The EA-activated wing reduced the maximum horizontal distance traveled to reach vertical flight ($\gamma =90^\circ$) by approximately four meters (17%) at 17 m/s (Fig. 4C) and increased maximum angular flight path velocity by 13 deg/s (18%) at 17 m/s (Fig. 4D).

## Discussion

It is well known that large-scale shape changes can improve efficiency and maneuverability for aircraft in flight^22^. Our findings suggest that constructing morphing wings to include feathers that behave similarly to those found in nature can effectively embed compliance into an airfoil structure, offering further efficiency boosts, while removing aerodynamic losses from feather gaps to bolster rapid direction change^21,37^. By comparing the feathered wing performance to the baseline wing and the similarly shaped rigid plate wing, we gained perspective on the influence of geometry and compliance on wing performance. The feathered wing offered a compliant trailing edge with and without gap formation, depending on the activation of EA. This generated aerodynamic effects that contrasted with the rigid plate wing to show the effect of trailing edge compliance while maintaining a gapless surface. Additionally, the voltage-driven activation of these adhesive feathers offers unique advantages for morphing wing flight.

Incorporating voltage-modulated EA into feather-based morphing designs can improve UAS adaptability for flying in complex and changing environments. EA feather fastening can temporarily lock the wing into a desired gapless shape during flight. Alternatively, shape change can be achieved by modulating the EA voltage with a high-frequency waveform, reducing friction to allow the feathers to slide over one another while maintaining contact (Fig. S3). This offers the adaptive benefits of wing morphing while mitigating the aerodynamic losses associated with feather gap formation. Improving maneuverability and efficiency opens the door for small UAS to enter domains currently unsafe for aircraft use, including forests and cities. This can aid first responders in search and rescue missions, as well as advance society toward smart city infrastructures to improve citizen safety and quality of life^44,45^.

## Methods

### Electrostatic adhesion

Electrostatic adhesive (EA) devices are found in a wide variety of applications, from semiconductor chucking systems to actuation systems and robotic end effectors^46–49^. In a typical EA device, an electrode is adhered to one side of a dielectric material. A second electrode acts as a braking surface between itself and the open face of the dielectric. Depending on the volume resistivity of the dielectric, there are two different regimes of electroadhesion: Coulombic and Johnsen–Rahbek (JR)^50^.

Most EA devices use a dielectric with high volume resistivity (≈1013–1018 Ω cm) that corresponds to Coulombic forces only. Under an applied voltage, the Coulombic EA force may be modeled as two parallel RC circuits in series due to the formation of an air gap between the dielectric and electrode. The resulting normal force scales quadratically with the applied voltage, where $A$ is the apparent contact area, ${\varepsilon }_{0}$ the permittivity of free space, d the dielectric thickness, g the gap distance, and ${\varepsilon }_{{\rm {d}}}$ and ${\varepsilon }_{g}$ are the dielectric and air gap permittivity^46,51–54^.

For a dielectric with ≈109–113 Ω cm, an additional attractive force is present, termed the Johnsen–Rahbek force (JR), and may also be modeled as two parallel RC circuits in series^50,54^. Here, due to the dielectric lower volume resistivity and migration of charge towards the electrode surface, much of the applied voltage is present at the micron-sized gap between the interfaces. Due to constricting surface asperities, the effective gap voltage is approximated as the applied voltage since the contact resistance is much greater than the bulk resistance of the dielectric. This produces a strong EA force, which only depends on the gap distance and dielectric constant (in this case, air)^46,50,54–58^. Again, the additional resulting normal force scales quadratically with the applied voltage. The total EA normal force, ${F}_{{{\rm{EA}}}}$, in this case given by the sum of the Coulomb, ${F}_{{{\rm{C}}}}$, and JR force, ${F}_{{{\rm{JR}}}}$,

2 $${F}_{{{\rm{C}}}}=\frac{A{\varepsilon }_{0}}{2}{\left(\frac{{\varepsilon }_{{\rm {g}}}{\varepsilon }_{{\rm {d}}}}{{\rm {d}}{\varepsilon }_{{\rm {g}}}+g{\varepsilon }_{{\rm {d}}}}V\right)}^{2},$$

3 $${F}_{{{\rm{JR}}}}=\frac{A{\varepsilon }_{0}}{2}{\left(\frac{{\varepsilon }_{{\rm {g}}}}{g}V\right)}^{2},$$

4 $${F}_{{{\rm{EA}}}}={F}_{{{\rm{C}}}}+{F}_{{{\rm{JR}}}}.$$

### Wing assembly

The wing airfoil is a Wortmann FX 60-126 airfoil (Fig. S4) which has been reported in previous work as a good fit among low Reynold’s number airfoils to a pigeon wing in gliding flight^26^. Spanwise sections were printed with a Markforged X7 3D printer out of Onyx filament which is a nylon polymer filled with short carbon fiber. Three sections ~151.7 mm in length were joined together to create an assembled span of ~455 mm.

A 1/4”–20 all-thread rod was placed through the quarter chord (32.5 mm rear of the leading edge) of the airfoil at the center thickness, providing spanwise bending stiffness. The all-thread rod extended to a load cell and motor mount for pitch control on the inboard side of the model. A baseline model (Fig. S4B) was used to compare against modified designs for which the rear 65 mm of the airfoil was removed (Fig. S4C, D). In two cases, the trailing edge was either replaced with a 73 mm chord 1.2 mm thickness 6061 Aluminum sheet (Fig. S4C) or a series of nine 55 mm in span “feathers” (Fig. S4D, E).

Biological feathers have been reported^59^ to have an elastic modulus of around 5–7 GPa measured by nano-indentation for pigeons and barn owls, which is higher than engineered plastics (2–3 GPa). To better match the stiffness of biological analogs, engineered feathers were 3D printed with continuous carbon fiber (CCF), which can have a tensile and bending stiffness on the order of 5–20 GPa depending on the fiber volume fraction^60^.

In this work, engineered feathers were printed with a target “rachis” dimension of 63.5 mm by 5 mm by 1.125 mm and a “flag” areal dimension of 63.5 mm by 55 mm at 2 mm wide. Onyx (white, Fig. S4F) paths of material were printed at a thickness of 0.125 mm (9 total layers), starting with two perimeter paths for all external walls at a width of 0.40 mm each. The total wall dimension then on all sides was 0.80 mm wide, which resulted in no space for the printer to put CCF reinforcement in the engineered feather flag; however, the rachis had three paths of CCF (blue, Fig. S4F). The feather had a target CCF volume percentage of 25–30% in the rachis, which provided 10–15 GPa longitudinal bending stiffness and comparatively lower torsional stiffness related to the lack of CCF in the flag.

**Fig. 5:**
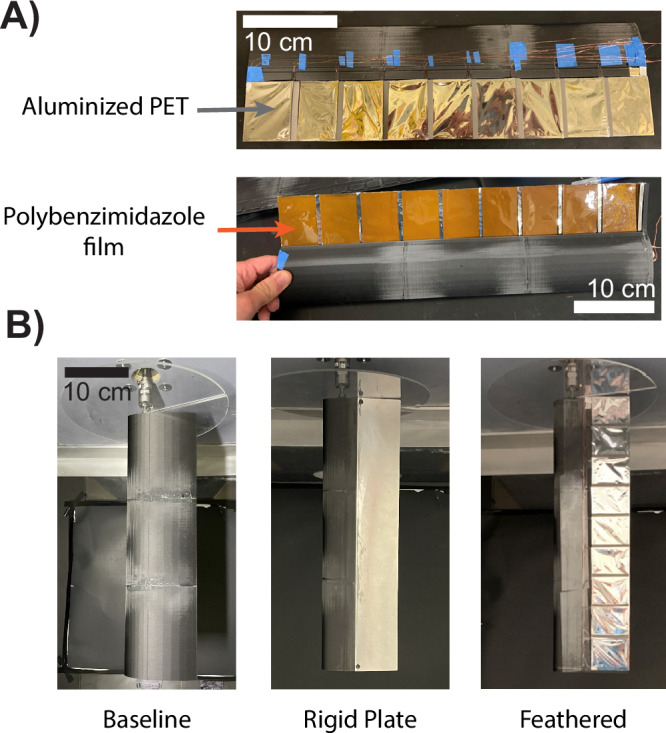
The feathered wing was constructed using electrostatic adhesive materials and compared against a baseline airfoil shape and rigid plate wing in a wind tunnel. **A** The top of each 3D printed feather was coated in an aluminized PET, and the bottom was coated with a polybenzimidazole film to generate coulombic and Johnsen-Rahbeck adhesive forces when subjected to high voltages (150−300 V). **B** The feathered wing was compared to a rigid baseline and plate wings, each mounted vertically in the wind tunnel with a baseplate on the mount side.

Polybenzimidazole (PBI) film (55 µm) and aluminized PET (12.5 µm) were attached to opposite sides of the “flag” region of the 3D-printed feather frames using double-sided adhesive tape (Fig. 5A). Nickel spray paint (MG Chemicals, Super Shield) was applied to one side of the PBI film prior to attachment to the feathers. For overlapping feather regions, both PBI and Al-PET were extended 5 mm past the feather edges to produce an overlapping area of ≈12 mm × 63.5 mm. Double-sided tape was first attached to both sides of the feather frame, trimmed to fit the frame, and then placed against the non-conductive side of a piece of Al-PET. The Al-PET was cut to fit the feather frame except for the noted 5 mm overhang, which overlaps the adjacent feather surface. On the PBI side, a 20 mm strip of single-sided copper tape was attached at the feather insertion, overlapping the double-sided tape (for electrical connection). The PBI film was attached in the same manner as the Al-PET by placing the frame tape-side down against the painted nickel surface on the backside of the PBI film. Finally, the PBI film was carefully cut to avoid damaging the Al-PET overhang, leaving a 5 mm overhang of PBI on the opposite side of the feather.

Electrical connections were made using 36 AWG magnet wire by physically stripping 1 cm of the enamel coating from the terminations using a razor blade. For the PBI side, the wire ends were soldered to the strip of single-sided copper tape, and the electrical connection was completed using conductive silver epoxy between the copper tape and nickel paint. Electrical connections to Al-PET were made by directly attaching the stripped wire ends to the aluminized surface using conductive silver epoxy. Note, other attachment methods to the aluminized PET surface, including conductive carbon tape, conductive copper tape, or mechanical connections, resulted in loss of connectivity over time due to degradation of the thin aluminum layer near the interface boundaries caused by electrical arcing.

During testing, since only one side of the Al-PET film is conductive, no shorting was observed through the PET film since the maximum applied voltage was well below the dielectric breakdown strength of PET (dielectric strength of PET ≈ 150–600 V/µm; electric field strength ≈24 V/µm at maximum voltage of 300 V). This field strength was sufficient for adhesive purposes but was not strong enough to produce significant deformation, and trailing-edge feather deflection due to EA activation was not observed during testing. The individually assembled feather “rachis” were press fit into slots along the main wing and permanently fixed using epoxy (DP125). Once the feathers were secured, the wires were bundled and placed into a slot along the wing surface, then covered with a strip of clear tape. DC voltage of 0–300 V was applied across the PBI and Al-PET interface using a Trek 10/10B-HS high voltage power supply controlled using a BK Precision 4063 function generator. The current draw was measured to be 0.3 µA at 300 V for 0.09 mW power draw for a single feather pair, or 0.012 mW/cm^2^, for a total power draw of 0.72 mW for eight overlapping regions in the whole wing assembly. Although we used a large laboratory-grade power supply and function generator to simplify experiment design, EA operation in aerial robotics can be achieved using much lighter electronics (~23 g) and a separate battery (~50 g) if designers prefer not to use the primary power source for the aircraft (Table S1)^61,62^.

Although experimentation was limited to the scale of the wing described, Eqs. (1)–(4) provide insight regarding the relationship between aerodynamic loading and EA strength as scale changes. Aerodynamic loading and EA attraction force increase linearly with surface area, suggesting the findings presented in this work would effectively scale with changes in wing and feather size. Additionally, power consumption will scale linearly with surface area according to 0.012 mW/cm^2^. Aerodynamic loads scale quadratically with flow velocity (Eq. 1) and EA attraction forces scale similarly with voltage (Eqs. (2)–(4)). This suggests that wing designs developed for flight at higher velocities can account for the increased loads by applying greater voltages to the EA system.

### Shear traction testing

PBI and Al-PET “flags” of the same dimensions as described above for the wing assembly were clamped to a custom-built test fixture for measuring shear EA forces (Fig. S3A). The same overlapping region as used in the wing assembly was used for all traction tests (12 mm × 63.5 mm). Traction tests were performed perpendicular to the long axis of the flags. To limit deformation of the polymer films and flags during testing, the entire 63.5 mm edge of each flag was firmly clamped to the test fixture. The flag surfaces were positioned parallel to one another (misalignment may induce peel moments, significantly lowering the peak traction force), then voltages of 0, 150, 200, and 300 V were applied using a function generator and high voltage power supply. The shear force required to separate the EA interface was measured using a CALT DYLY-103 5 kg load cell (0.03% accuracy) at a separation rate of 100 mm/min. Three trials were performed at each voltage (Fig. 1F).

Using the same experimental setup (Fig. S3A) we tested how shear traction varied at three separation speeds (160, 400, and 800 µ/s) and a range of sine wave voltage frequencies (1 Hz–10 kHz) oscillating between 0 and 300 V as well as when held constant at a DC voltage of 150 V. These tests supported the concept of using EA feathers for morphing and can inform the control process for voltage modulation during wing shape change. For instance, we found that at low speed, shape change, the high frequency and constant voltage EA produce a stick-and-slip behavior (Fig. S3B). Additionally, the low-frequency EA experienced full disconnection during sliding at the higher separation speeds (400 and 800 µ/s). Higher speed shape changes show that the frequency modulated EA voltages provide a lower peak traction than the constant DC EA (Fig. S3C), but maintained a stronger connection during sliding (Fig. S3D).

### Wind tunnel testing

Experiments were performed in the DEVCOM Army Research Lab’s low-speed, recirculating wind tunnel located at Aberdeen Proving Ground. The half-span wing models were mounted vertically from the ceiling to limit potential aeroelastic instability, although none was observed during testing (Fig. 5B). A circular acrylic baseplate was fixed to the root of each wing, but the tip of the wing was left free to include 3-dimensional aerodynamic effects in our measurements. The wing root and base plate were mounted approximately four inches from the test section ceiling to ensure the entire wing remained outside of the wind tunnel boundary layer during testing. A 6-axis ATI Nano 25 load cell was connected to the wing at the ¼ chord location on the root side of the wing. This load cell was mounted to a servo motor used to control the angle of attack of the model, sweeping upward from −5° to 20°, pausing at each angle of attack to collect aerodynamic and torque measurements, and then repeating the measurements sweeping downward from 20° to −5°. Data collection occurred at 1000 Hz for 5 s at each tested angle of attack. A 50 Hz low-pass filter was applied to the raw data to remove excess noise measured at the 60 Hz frequency. The first second of data was removed from each collected data set to allow for the signal to settle, resulting in *N* = 4000 measurements per data collection period. This procedure was repeated 4 times for each wing at each flow velocity, resulting in *N* = 32,000 data points at each angle of attack, velocity, and wing condition. After data collection, the load cell measurements associated with the *x* and *y* axes were rotated by the appropriate angles of attack to ensure proper lift and drag measurements were recorded. The servo motor was controlled by a Galil DMC-4020 controller, which leveraged a PID controller for motion control. A 4000 count per revolution encoder was used to track the position of the model, with a resulting angular resolution of 0.09°. Throughout the experiment, the servo motor held the model at the prescribed angle, but in some cases, where unsteady loading was present, some jitter in the encoder was observed. This was always on the order of (±)1 encoder count from the set point, resulting in an angular uncertainty in the prescribed angles of 0.18°. Models were aligned to the flow using a laser line projected from the floor of the wind tunnel. Based on the thickness of the laser line on the model, we assess that the uncertainty in *α* = 0° to be approximately (±)0.25°. The maximum uncertainty in the model angle of attack for any given case was therefore (±)0.3°, with most of that uncertainty in model mounting.

Experiments were conducted for three set airspeeds: *U* = 11.5, 14.3, and 17 m/s. Prior characterization of the wind tunnel turbulence levels showed that for these airspeeds, the turbulence intensity was less than 0.1% of the freestream speed. There was some variation in model chord length, with models ranging from 0.125 to 0.132 m. While this was accounted for in the calculation of the lift, drag, and pitching moment, it does generate some minor variability in the Reynolds number. At the lowest tested airspeed, the Reynolds number variation was approximately ±3000 (Re ≈ 97,000–103,000), and increased slightly to ±4000 (Re ≈ 144,000–152,000) at the highest flow speed. In this range of Reynolds numbers, that level of variation was unlikely to have any major aerodynamic effects and proper normalization of coefficients (*C*_L_, *C*_D_, *C*_m_) was deemed sufficient to nullify the variability in the model sizes. During testing, no torsional deflection was observed for any of the tested wings, and bending deflection was minimal (<5°) and consistent across tested wings. Since only longitudinal aerodynamic forces and moments were considered, this small deflection was considered acceptable. Uncertainty in these coefficients was defined using 95% confidence intervals based on multiplying the standard deviation of the collected load cell measurements by 1.96.

### Maneuver simulation

To illustrate how EA changed the maneuverability of the feathered wing, we developed a 2D flight dynamics model. In Python, we fit a linear regression model to the data to estimate the coefficients of lift, $\hat{{C}_{L}},$ and drag, $\hat{{C}_{{\rm {D}}}}$ (Fig. S2) of the active EA (${\hat{C}}_{{\rm {L}}}:\,{R}^{2}=0.994$, ${\hat{C}}_{{\rm {D}}}:\,{R}^{2}=0.864$) and passive feathered wing (${\hat{C}}_{{\rm {L}}}:\,{R}^{2}=0.995$, ${\hat{C}}_{{\rm {D}}}:\,{R}^{2}=0.847$) provided the angle of attack, $\alpha$, and velocity in the wind frame, $U$,

5 $${\hat{C}}_{{ {L}}}={A}_{0}+\,{A}_{1}\alpha +{A}_{2}U+{A}_{3}\alpha U+{A}_{4}{\alpha }^{2}+{A}_{5}{U}^{2},$$

6 $${\hat{C}}_{ {{D}}}={B}_{0}+\,{B}_{1}\alpha +{B}_{2}U+{B}_{3}\alpha U+{B}_{4}{\alpha }^{2}+{B}_{5}{U}^{2},$$

where ${A}_{n}$ and ${B}_{n}$ are the *n*th coefficient of each model and $n=0$ represents the intercept of each model. Angle of attack, $\alpha$, and flow velocity, $U$, were the basis of each independent variable^63^. It should be noted that pitch rate, *q*, was not included in the ${\hat{C}}_{{\rm {L}}}$ and ${\hat{C}}_{{\rm {D}}}$ calculation. Dynamic pitching experiments were performed in the wind tunnel for this purpose; however, we found the linear models produced negligible coefficients for the value, and their inclusion reduced accuracy.

**Fig. 6:**
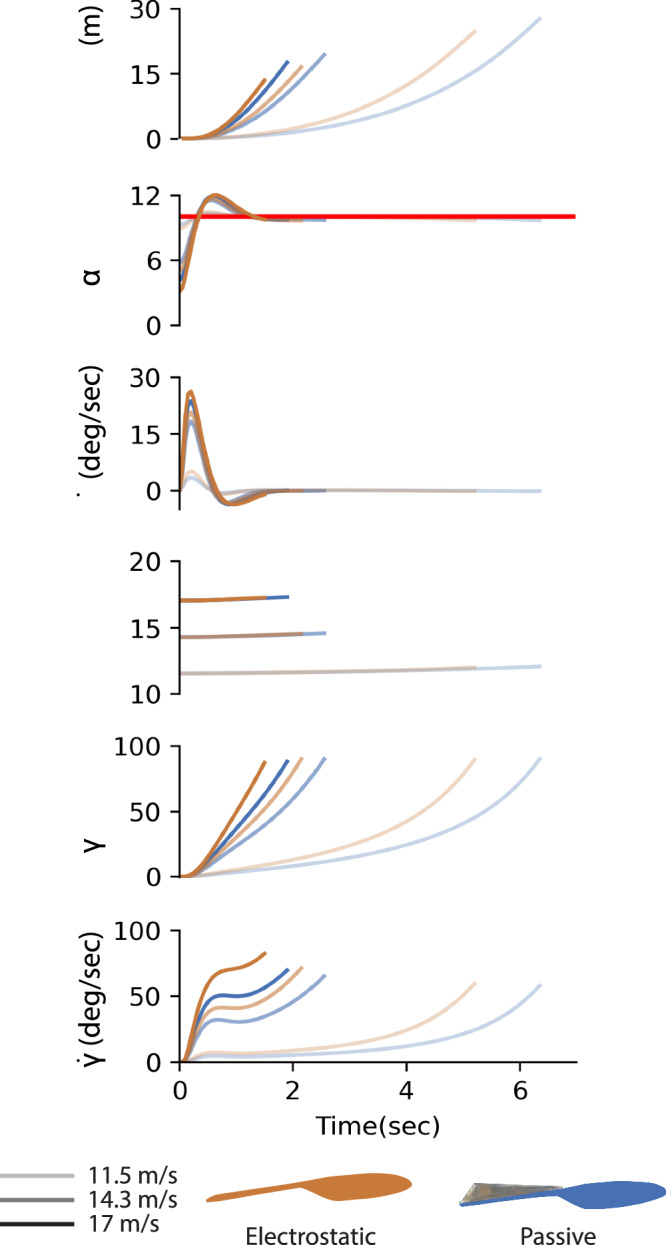
A PID controller used angle of attack feedback to adjust pitch rate and provide a fair comparison between electrostatic (orange) and passive feathered wings (blue) during the pitch-up maneuver. We found close agreement between the electrostatic and passive wings’ angles of attack (α) when approaching and maintaining the target angle (*α*_target_ = 10°; red line) for each flight speed (*U* = 11.5, 14.3, 17 m/s; distinguished by increased opacity). The electrostatic wing consistently showed more aggressive flight, achieving a more rapid increase in flight path angle, $\gamma$.

We modeled the point-mass flight dynamics with a constant discrete timestep size, ${dt}$, of 0.05 seconds, using the wind-frame state variables velocity, ${U}_{t}$, and flight path angle, ${\gamma }_{t},$ at each timestep, $t$^64^$.$ (Fig. 6). The state variables were calculated using

7 $${U}_{t+1}=\frac{1}{m}\left(\left({T}_{t}\cos \left({\alpha }_{t}\right)-\hat{D}-m\,g\sin \left({\gamma }_{t}\right)\,\right){dt}+{U}_{t}\right),$$

8 $${U}_{t+1}{\gamma }_{t+1}=\frac{1}{m}\left(\left({T}_{t}\sin \left({\alpha }_{t}\right)+\hat{L}-m\,g\cos \left({\gamma }_{t}\right)\right){dt}+{\gamma }_{t}\right),$$

where the predicted lift, $\hat{L}$, and drag, $\hat{D}$, were determined by inverting the relationship presented in Eq. (1),

9 $$\hat{D}=\frac{1}{2}\rho {{U}_{t}}^{2}\,S\,{\hat{C}}_{D},$$

and,

10 $$\hat{L}=\frac{1}{2}\rho {{U}_{t}}^{2}\,S\,{\hat{C}}_{L}.$$

Velocity was held constant velocity through the pitching maneuvers (11.5, 14.3, 17 m/s) by throttling the thrust, ${T}_{{\rm {t}}}$, at each timestep,

11 $${T}_{{\rm {t}}}=\frac{\,\left(\frac{1}{2}\rho \,{{U}_{{t}}}^{2}{S}{\hat{C}}_{{\rm {D}}}\right)+{m\; g}\sin \left({\gamma }_{{\rm {t}}}\right)\,}{\cos \left(\alpha \right)}.$$

To provide a more intuitive representation of the flight trajectory, we transformed the wind-frame state variables into the Earth-frame for each timestep as well using

12 $${x}_{t+1}={U}_{t+1}\cos \left({\gamma }_{t}\right){dt}+{x}_{t},$$

and

13 $${h}_{t+1}={U}_{t+1}\sin \left({\gamma }_{t}\right){dt}+\,{h}_{t},$$

where ${x}_{t}$ and ${h}_{t}$ represent the horizontal and vertical distance traveled during the maneuver.

Note that the pitching moment was not considered in these equations based on the assumption that the pitch rate, $q$, would be adequately controlled by an alternative control surface, such as an elevator, to limit the focus of these simulations to the change in performance achieved directly from the use of EA on the feathered wing, as opposed to its control dynamics. The pitch-up maneuver was achieved using a discrete PID controller to create a change in pitch rate at each timestep, ${\Delta q}_{t}$,

14 $${\Delta q}_{t}=\,{K}_{{ {p}}}\left({\varepsilon }_{t}+\frac{1}{{T}_{{\rm {i}}}}\mathop{\sum }\limits_{j=0}^{t}{\varepsilon }_{j}+{T}_{{ {d}}}\frac{{\alpha }_{t}-{\alpha }_{t-1}}{{dt}}\right),$$

where the signal error, $\varepsilon$, was calculated at each timestep as the difference between the current angle of attack, ${\alpha }_{t}$, and the target angle of attack, ${\alpha }_{{{\rm {target}}}}=10^\circ$,

15 $${\varepsilon }_{t}=\,{\alpha }_{t}-{\alpha }_{{{\rm {target}}}},$$

and the proportional gain (${K}_{{\rm {p}}}=40$), integration time (${T}_{{\rm {i}}}=0.5$), and derivative time (${T}_{\rm {{d}}}=0.1$) were tuned by hand^65^. The change in pitch rate resulted in a new pitch, ${\theta }_{t+1}$, and angle of attack, ${\alpha }_{t+1}$,

16 $${\theta }_{t+1}=\,{\theta }_{t}+\frac{1}{2}\left(2{q}_{t}+{\Delta q}_{t}{dt}\right){dt},$$

17 $${\alpha }_{t+1}=\,{\theta }_{t+1}-{\gamma }_{t+1},$$

for use in the lift and drag calculations for the following timestep (Eqs. (5)–(8)). Additionally, error was propagated through maneuverability performance metrics using the minimum and maximum bounds of the 95% confidence intervals according to the aerodynamics linear regression models, ${\hat{C}}_{{\rm {L}}}$ and ${\hat{C}}_{{\rm {D}}}$. Finally, further analysis of the flight trajectories showed that the angle of attack, $\alpha$, remained below stalling conditions and small $\dot{\alpha }$ was satisfied, as determined by

18 $${\Omega }^{* }=\frac{\dot{\alpha }c}{2U} < 0.01,$$

where $\dot{\alpha }$ is in radians per second and $c$ is the wing chord length ($c=0.127$ m).

## Supplementary information

Transparent Peer Review file

Supplementary Information

## Data Availability

Raw data is available on the public figshare repository: https://figshare.com/articles/dataset/Electrostatic_adhesion_mitigates_aerodynamic_losses_from_gap_formation_in_feathered_wings/29185271?file=54935921. Additionally, box-and-whisker plots representing the full distributions of raw C_L_ and C_D_ data are available in the Supplementary Materials (Fig. S10).

## Code availability

Code used for data analysis is available on the public github repository: https://github.com/kevpatha/EA_feathers.
