# Supplementary material for: Electrostatic adhesion mitigates aerodynamic losses from gap formations in feathered wings
Source: Commun Eng. 2025 Oct 13;4:178. doi: 10.1038/s44172-025-00452-z (PMC12518682; doi:10.1038/s44172-025-00452-z)
Supplement: Supplementary file 2 — Supplementary Information [file 44172_2025_452_MOESM2_ESM.pdf]

Supplementary Materials for

Electrostatic adhesion mitigates aerodynamic losses from gap formations in feathered wings

Kevin PT Haughn, Jeff T Auletta, John T Hrynuk, Todd C Henry
Corresponding author: kevin.p.haughn.civ@army.mil

The file includes:

Supplementary Text
Figs. S1 to S10
Table S1
Supplementary References

Supplementary Text

Drag and Moment for all velocities

The coefficient of lift, C_L, polar was the only longitudinal aerodynamic force presented for all three velocities. We have included the analogous coefficient of drag, C_D, and coefficient of moment, C_m, polars here (fig S1).

Linear Regression Modeling

The linear regression models used for simulating the pullup maneuvers in Figure 4, we used ordinary least squares model that accurately approximated the C_L and C_D throughout the flight envelope of the simulated maneuvers, including velocities ranging from 11.5 m/s to 17 m/s, and angles of attack prior to stall (-5° to 12°) (fig. S2). Figure 6 shows that the simulated flight maneuvers remained within these ranges, supporting the trustworthiness of the findings stemmed from these simulations. The high R^2 values further suggest that these models offer a strong fit to the measured data, and low RMSE values quantified the uncertainty within the C_L models C_L (Passive: $R^2 = 0.995$, RMSE = 0.023; EA: $R^2 = 0.994$, RMSE = 0.028) and C_D models (Passive: $R^2 = 0.847$, RMSE = 0.010; EA: $R^2 = 0.864$, RMSE = 0.028).

Metrics and Statistical Modeling

We defined performance metrics that fell into two categories, maximums (C_{Lmax} and L/D_{max}) and α-relationships ($C_{L\text{-}\alpha}$). The maximum-based metrics were derived from a maximum average value at a single angle of attack. However, for each wing, the angle of attack was not always consistent between each sweep direction (upward and downward) and iteration. Therefore, after determining the max-value angle of attack for each respective sweep direction and iteration, we incorporated all collected measurements from the appropriate individual angles of attack for each iteration and sweep direction for statistical analysis.

The α-relationship based metrics, $C_{L\text{-}\alpha}$, required force and moment measurements from several angles of attack for each sweep direction and iteration. We limited the definition of these metrics to fall within the linear flight regime ($\alpha \leq 10°$).

We used the linear mixed effects (LME) model function in the statsmodels Python library to perform statistical analyses on our experimentally measured performance metrics while accounting for the random measurement behavior that naturally occurred between experimental sweep directions (upward and downward) and four measurement iterations. The statsmodels package uses the equation format,

$$Y_{ij} = \beta_0 + \beta_1 X_{ij} + w_{0i} + w_{1i} X_{ij} + \epsilon_{ij}, \qquad (eqn. S1)$$

to solve for fixed effect parameters, β_0 and β_1, and random effect parameters, w_{0i} and w_{1i}, where X_{ij} is a covariate for the jth measured response, Y_{ij}, of subject i, with independent errors, ϵ_{ij}.

LME follows the assumption of linearity that can be satisfied analyzing models' residual distributions for normality. Provided the large size of the datasets from which we modeled the desired metrics and their relationships to velocity (N of order 10^4-10^6), central-limit-theorem allowed us to assume Gaussian errors appropriate for the linear mixed models used here (*1, 2*). Additionally, to promote data normality, we use 1.5×IQR (interquartile range) bounds on our

measurements to reduce the impact of outliers. Still, we checked for residual normality using the Shapiro-Wilks statistic (W), while neglecting the associated p-value since the significance metric is known to be inaccurate for Shapiro-Wilks tests on large datasets. The Shapiro-Wilks statistic predicts greater normality with values closer to 1. Additionally, we considered uncertainty within the model by reporting accuracy levels using root mean squared error (RMSE).

We found that using the most simplified LME models for analyzing the $C_{L\text{-}max}$ producing individual models for each wing (baseline, plate, passive, and active) and velocity (11.5 m/s, 14.3 m/s, 17 m/s) offered the greatest accuracy (RMSE < 1%) and achieved approximately normal residual distributions (W > 0.95) (figs. S5), which further supports the predictions and 95% confidence intervals as trustworthy (Fig. 2D). The $C_{L\alpha}$ LME produced accurate results, with RMSE< 0.015 for each tested velocity. Given the linear range of values used for the modeling was C_L~0.3 on average, this RMSE is less than 5% error. Additionally, the Shapiro-Wilks statistics were approximately 1 for each model, suggesting normal distributions (fig. S6), supporting trustworthy predictions and statistics for the $C_{L\alpha}$ predictions (Fig. 2F).

Calculating the relationship between C_{Lmax} and $C_{L\text{-}\alpha}$ with velocity required the inclusion of velocity interactions within the LME, thus increasing modeling complexity. Still each model achieved RMSE ~ 0.014 (fig. S7), resulting in less than 2% error for the C_{Lmax}-velocity relationship (Fig. 2E) and less than 5% error for the $C_{L\text{-}\alpha}$-velocity relationship (Fig. 2G). Additionally, each residual distribution produced Shapiro-Wilks statistics greater than 0.97, suggesting normality and therefore trustworthy modeling.

Again, we found that using the most simplified LME model, individually considering each wing-velocity combination, produced accurate (RMSE < 2%) and approximately normal residual distributions (W > 0.98) for the L/D_{max} metric (fig. S8), providing trustworthy individual points and statistics for Figure 3B. Incorporating interactions between L/D_{max} and velocity maintained accurate modeling with RMSE less than 5% of the used L/D_{max} measurements and approximately normal residual distributions (W=0.99) (fig. S9).

Supplementary Figure 1

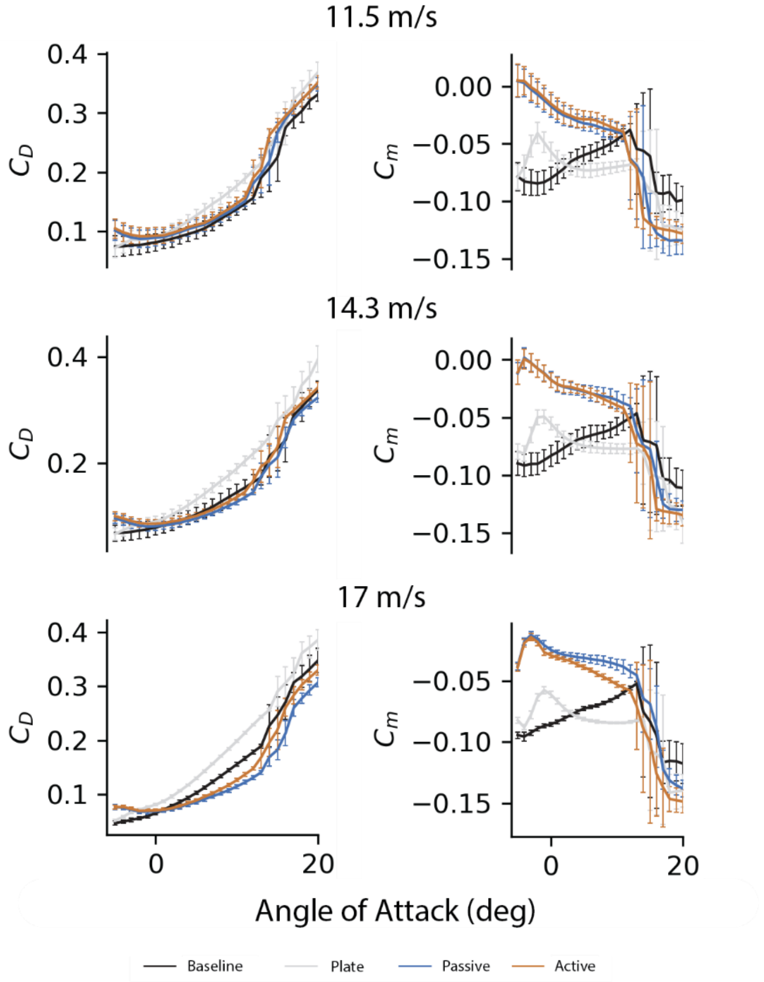

Wing compliance showed an impact on drag as velocity increased, and geometry showed impact on stability. A) the coefficient of drag for the compliant feathered wing remained low as velocity increased, but increased for the rigid wings. B) Slopes for each wing remain relatively similar between velocities, but the feather like airfoil shapes are negative, whereas the engineered airfoil is positive. Gap formation between feathers, which occurs at higher velocities, reduced slope steepness.

Supplementary Figure 2.

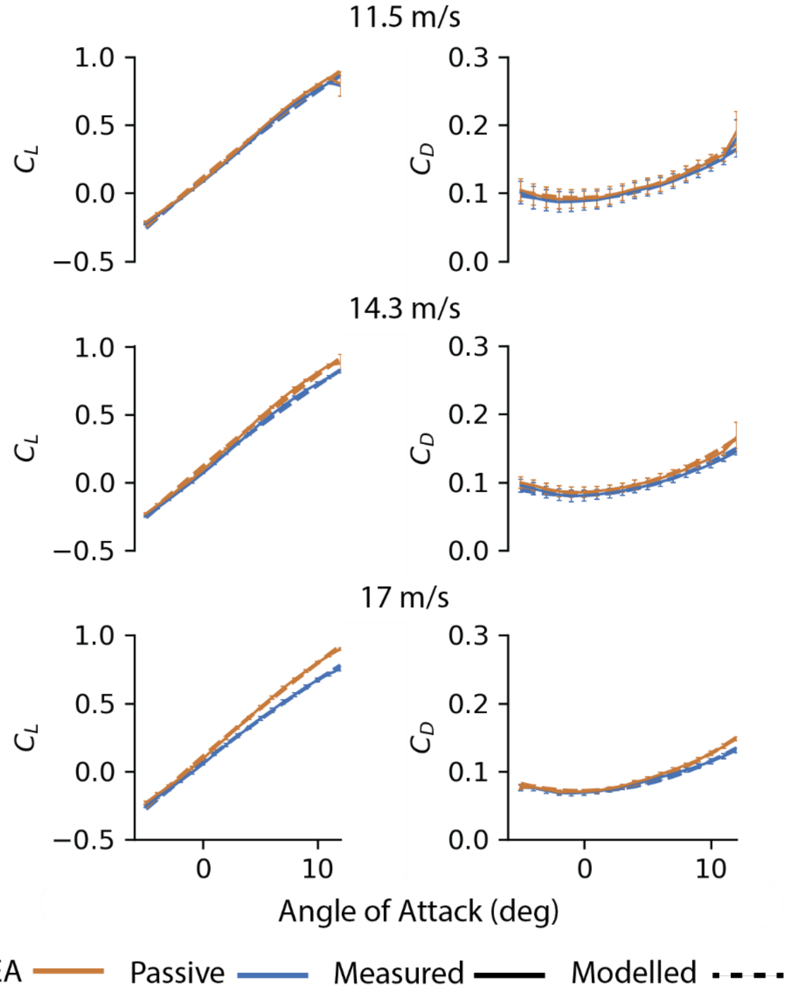

The linear regression models accurately predict the C_L (Passive: $R^2 = 0.995$, RMSE = 0.023; EA: $R^2 = 0.994$, RMSE = 0.028) and C_D (Passive: $R^2 = 0.847$, RMSE = 0.010; EA: $R^2 = 0.864$, RMSE = 0.028) for measured velocities (11.5 m/s, 14.3 m/s, and 17 m/s) and angles of attack prior to stall, ranging from -5° to 12°.

Supplementary Figure 3

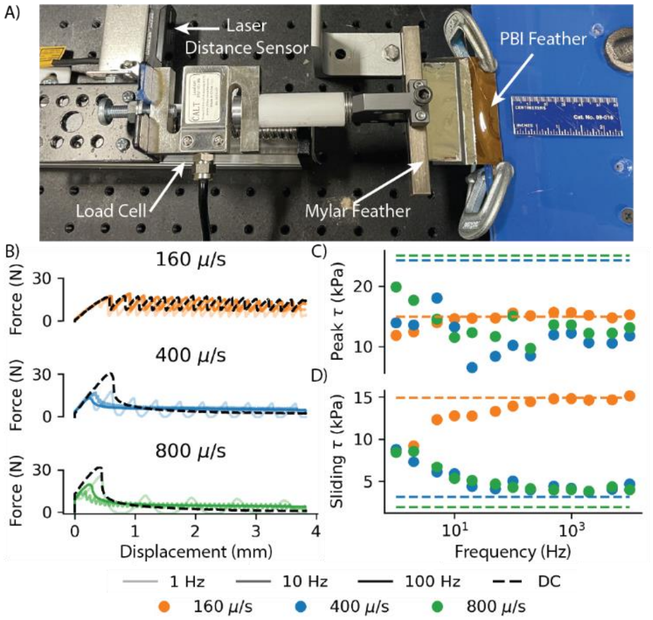

Shear traction tests showed effects of voltage frequency and morphing speed on shear traction. (A) We used a CALT DYLY-103 5 kg load cell (0.03% accuracy) to measure the force required to separate the feathers under shear loading during three constant separation speeds (160 μ/s: orange; 400 μ/s: blue; 800 μ/s: green) and a range of sine wave voltages oscillating between 0V and 300V at frequencies ranging from 1 Hz and 10 kHz. Feathers are 63.5 mm long, beginning with 20 mm of overlap. (B) As sine wave voltage frequency increased, the load response behaved more similarly to constant DC voltage (150 V; dashed black line). The slowest separation speed produced a “stick-and-slip” behavior. A similar “stick-and-slip” behavior was observed at the faster separation speeds for the lower voltage frequency cases, where enough relative time was spent at a low voltage to produce negligible friction force, suggesting the feathers were effectively disconnected. The high frequency and constant voltage cases did not experience the stick-and-slip behavior at faster separating speeds. Instead, these tests began with gradual build in force until they achieved a high static friction peak, followed by a lower kinetic friction force. (C) Initial peak traction grew to be similar to the constant DC voltage when separating at 160 μ/s as frequency increased but decreased to nearly half the DC peak shear traction, τ, when operating at high voltage frequencies during the two faster separation speeds. (D) The peak traction measured during sliding (peak traction at sliding positions greater than 2 mm) illustrates the force required to continuously slide the feathers after initial sliding has begun. Similar sliding traction was achieved for each sliding speed at low frequencies but quickly deviated as frequencies increased. Shear traction increased for high frequency and DC voltage to peak traction levels when sliding at 160 μ/s. Sliding friction reduced as frequency increased for the faster sliding speeds, but didn’t reach values as low as the DC case. This difference was largest for the fastest sliding speed.

Supplementary Figure 4

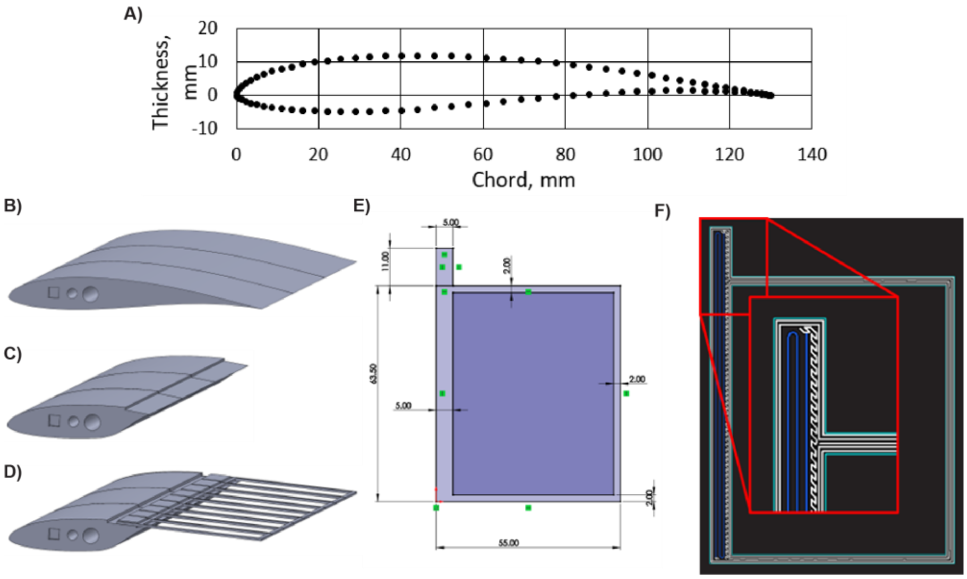

Each wing was constructed using a Markforged X7 3D printer. (A) We used a Wortmann FX 60-126 as the initial airfoil shape for each wing. (B) The baseline wing was entirely 3D printed, whereas the (C) rigid plate wing and (D) feathered wing included only the leading portion of the baseline airfoil shape, using an aluminum plate or 3D printed feathers for the respective trailing edges. (E) Each feather was printed using a nylon base material in a flag-like construction with a 55 mm X 63.5 mm planform shape. (F) The rigidity of the feathers was tuned using continuous carbon fiber (blue), including three paths within the rachis to increase stiffness.

Supplementary Figure 5

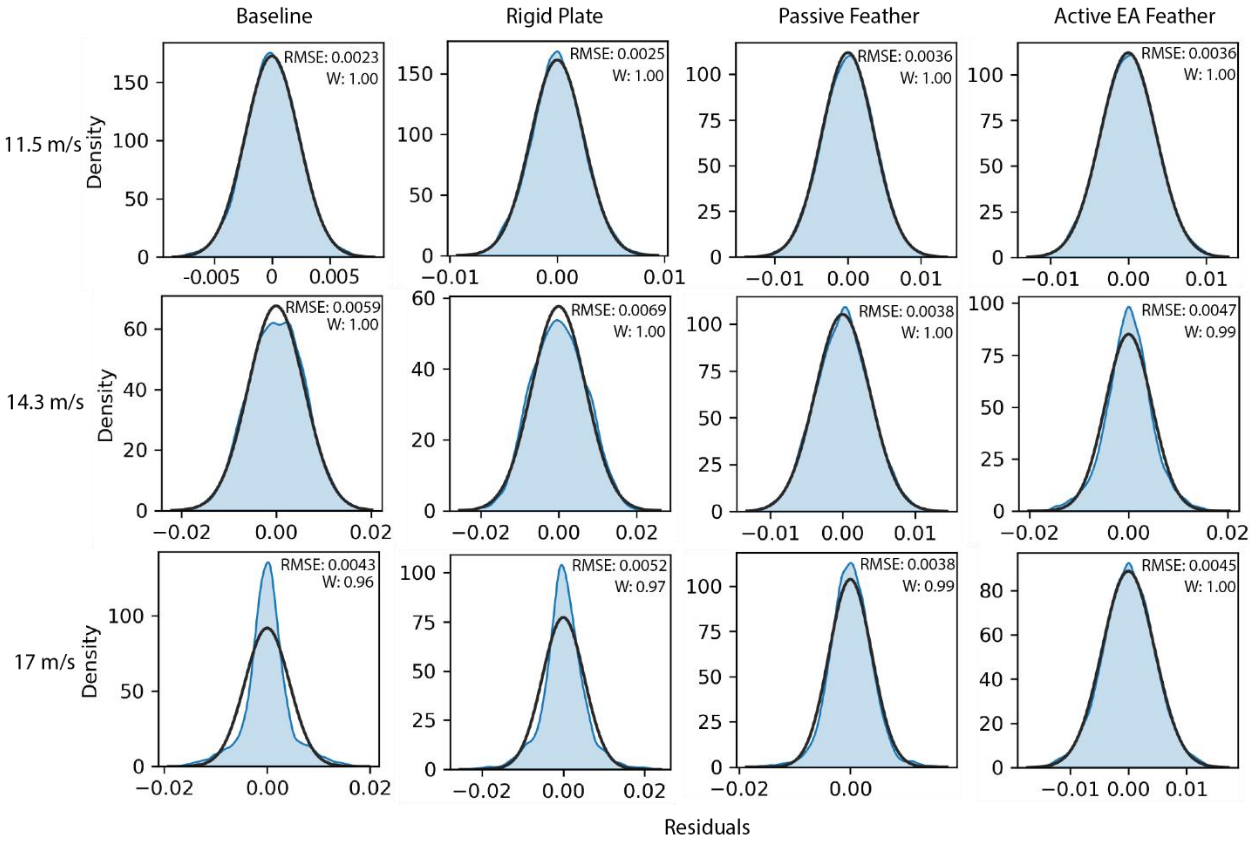

The linear mixed effects models used to estimate the maximum C_L for individual wing-velocity combinations produced accurate (RMSE < 1%) and approximately normal residual distributions (W > 0.95), supporting statistical results as trustworthy.

Supplementary Figure 6

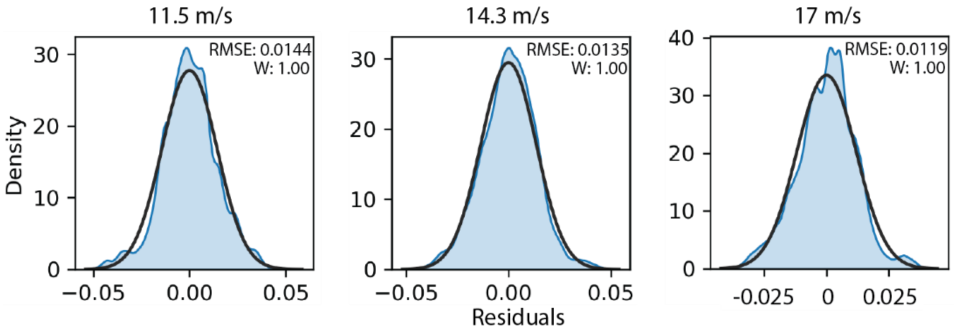

The linear mixed effects models combining each wing type at individual velocities produced accurate (RMSE ~ 0.01) and approximately normal residuals (W ~ 1), supporting statistical results as trustworthy

Supplementary Figure 7

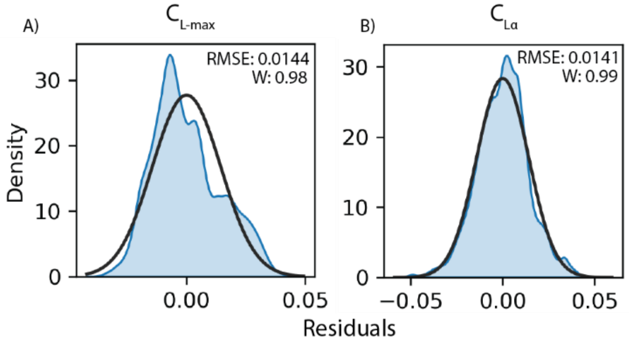

The linear mixed effects models used to consider the A) CL_{max}-velocity interaction and B) CL_{α}-velocity relationships were accurate (RMSE ~ 0.014) and approximately normal residuals (W > 0.97), supporting statistical results as trustworthy.

Supplementary Figure 8

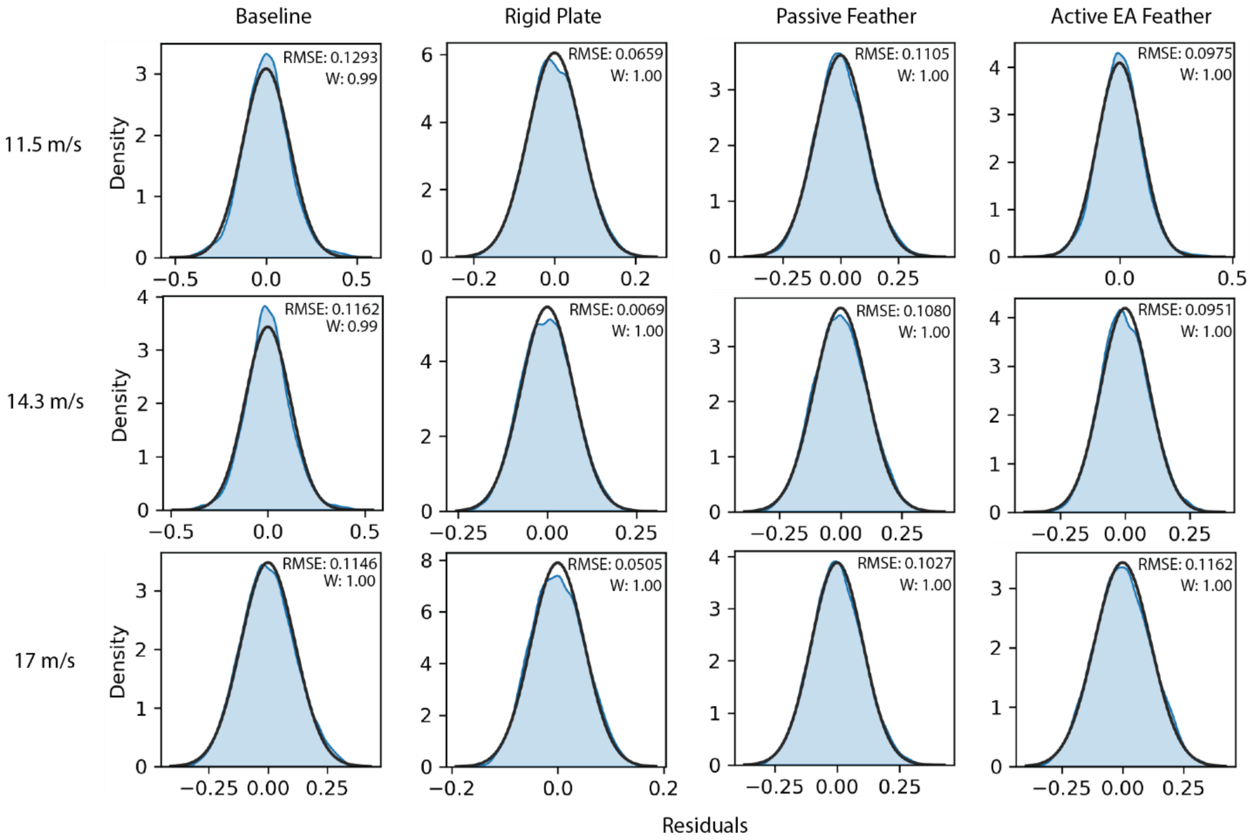

The linear mixed effects models used to estimate the maximum L/D ratio for individual wing-velocity combinations produced accurate (RMSE < 2%) and approximately normal residual distributions (W > 0.98), supporting statistical results as trustworthy.

Supplementary Figure 9

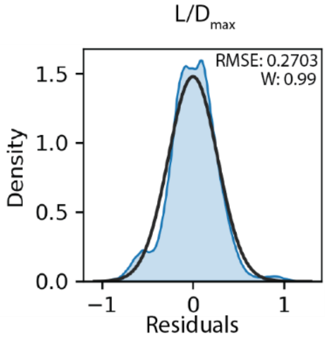

The linear mixed effects models used to consider the L/D_{max}-velocity interaction relationships were accurate (RMSE ~ 0.27) and approximately normal residuals (W = 0.99), supporting statistical results as trustworthy.

Supplementary Figure 10

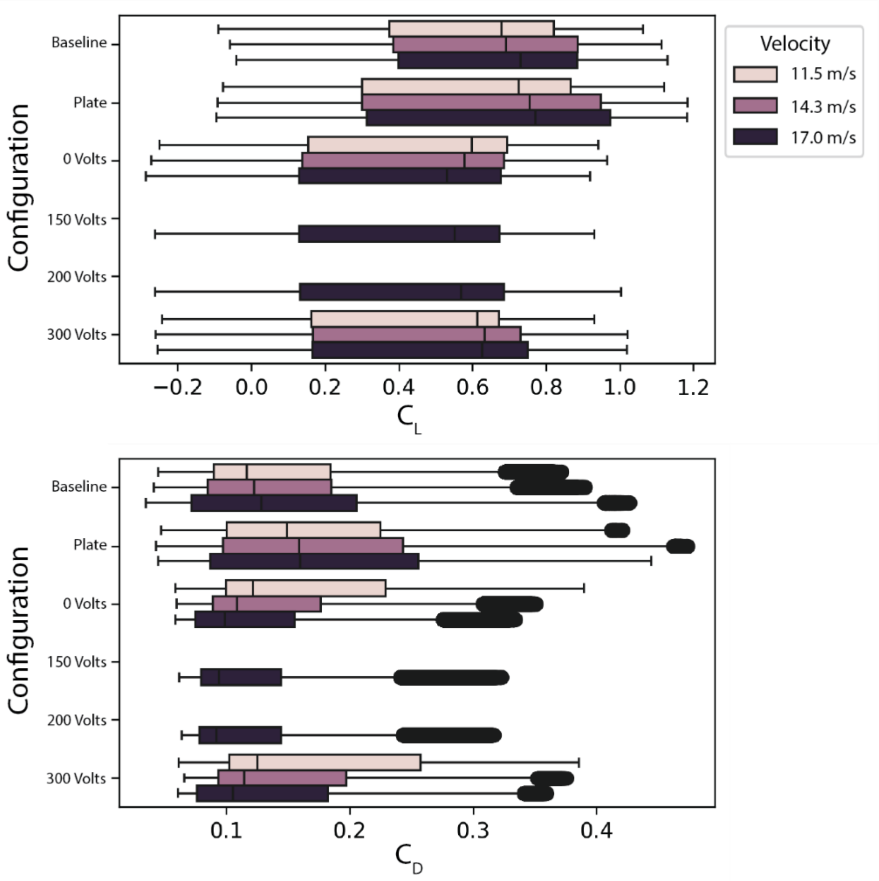

Box plots show the raw coefficient of lift (C_L) and coefficient of drag (C_D) data distributions for each configuration (baseline, flat plate, and feathered wings with 0, 150, 200, and 300 volts) at each flow velocity (11.5 m/s, 14.3 m/s, 17.0 m/s). The center vertical line represents the median, the edges of the box represents the first and third quartiles, and the whiskers extend outward to values of 1.5 times the inter quartile range. Outliers are illustrated as circles (seen overlapping in the C_D plot).

Supplementary Table 1

The mass of the electronics needed to control and amplify the voltage supplied for electrostatic adhesion sum to 22.33 grams without considering a power source. This assumes power would be pulled from the primary aircraft battery. For a system fully independent of the aircraft's power system, an additional mass of 48.34 grams would account for the needed battery.

Part	Mass (g)
Diode	0.13
Capacitor	0.57
IRF 4905	2.11
680μH inductor	0.89
Lipo shim	3.04
A20P-5(2kv)	3.91
Arduino Micro	6.68
PCB	5.00
Sub-total	22.33
Battery	48.34
Total	70.67

Supplementary References

1. U. Knief, W. Forstmeier, Violating the normality assumption may be the lesser of two evils. *Behavior research methods* **53**, 2576-2590 (2021).
2. T. Lumley, P. Diehr, S. Emerson, L. Chen, The importance of the normality assumption in large public health data sets. *Annual review of public health* **23**, 151-169 (2002).
