# Supplementary material for: Electrostatic adhesion mitigates aerodynamic losses from gap formations in feathered wings
Source: Commun Eng. 2025 Oct 13;4:178. doi: 10.1038/s44172-025-00452-z (PMC12518682; doi:10.1038/s44172-025-00452-z)
Supplement: Supplementary file 1 — Transparent Peer Review file [file 44172_2025_452_MOESM1_ESM.pdf]

This file contains all reviewer reports in order by version, followed by all author rebuttals in order by version.

Attachments originally included by the reviewers as part of their assessment can be found at the end of this file.

nature portfolio

Peer Review File

Learning Aerodynamics for the Control of Flying Humanoid Robots

Corresponding Author: Mr Antonello Paolino

Version 0:

Reviewer comments:

Reviewer #2

(Remarks to the Author)
The authors presented a paper that models a humanoid robot with jet packs to fly. The paper focuses on a system-level assessment of the dynamics and the neural network-based controller. The robot is not shown as a physical system, other than in wind tunnel experiments. An assessment and discussion of the overall architecture and flight behavior are presented. The paper is well written. I have the following comments:

1. The paper mentions that new methods in fluid dynamics have been developed or need to be developed, but it's not clear which methods these are. From the description, it seems that existing methods in CFD and wind tunnel analysis have been used without much innovation in the fluid dynamics part. It would be good if this could be clarified.
2. The assumption that the tests are Reynolds number independent is not well justified. For example, the appendix mentions a Reynolds number of more than 100,000; however, this is for the geometry of the head and at 17 m/s. Smaller parts of the body, such as the legs and arms, especially at lower speeds, could be in the range of 10,000 Reynolds number, which might affect the transition regime from laminar to turbulent. In this regime, their dynamics become much more complex and could therefore influence the robot's behavior and the forces interacting with the robot significantly. If a full Reynolds-dependent model cannot be developed or presented, then at least a detailed discussion of these effects and the effects on flight behavior would be very helpful.
3. The wind tunnel experiments showed the drag force; however, the graph does not show the lift force. When building a flying humanoid, it seems reasonable to assume that lift forces are also being used and benefited from. I would suggest having a discussion on this part and also demonstrating the measurements in lift force. It is also not very clear why the particular attitude of the robot has been chosen, as depending on the flight speeds, the attitude might change significantly, and therefore the forces and overall flow behavior might also differ greatly.
4. It is not clear how the interaction with the jet fluid flow interferes with the overall fluid dynamics of the human body. During flight, and especially around hover conditions, it seems very reasonable that the jet is a dominating fluid influence and therefore can change the dynamics of the body of the robot significantly. How can this be modeled? What are the velocities and forces involved, and how could an overall dynamic model for the behavior of the robot be developed in such a case?
5. Moreover, it is not clear whether a full aerodynamic model is needed if the dynamic forces are treated like a disturbance. Can the control deal with the forces without actually knowing the detailed forces acting on the robot and having a model to estimate them? Potentially, and especially around hover conditions, their dynamics might not be very important. If the flight speed being reduced is an option, then perhaps the overall problem would be simplified a lot. If the flight speed is intended to be kept high (as in the presented paper) then a different robot body would need to be developed that is more suitable for flight.

If these comments can be addressed, then the paper can be a valuable contribution to the literature and is a very exciting robot concept.

Reviewer #3

(Remarks to the Author)
This paper describes a jet-powered humanoid robot optimized for jet engine integration. Hardware modifications were made to the humanoid robot for wind tunnel experiments to enable precise measurement of aerodynamic forces and surface pressures. A comprehensive approach using both classical and learning techniques is provided for modeling and controlling the aerodynamic forces. These research findings offer new ideas and methods for related technical research on humanoid robots.

The main contributions of this paper encompass three aspects:

① The mechanical design of iRonCub-Mk1, a jet-powered humanoid robot optimized for jet engine integration;
② Hardware modifications to the humanoid robot for wind tunnel experiments, enabling precise measurements of aerodynamic forces and surface pressures;
③ The provision of a comprehensive method for modeling and controlling aerodynamic forces using classical and learning techniques.

The paper describes some fundamental principles, but there are contradictions and a lack of solutions to key issues. For detailed problems, please refer to the comments.

Regarding contribution ① several key design issues require explanation and verification:
1. The legs and waist of the robot in the current design do not demonstrate their corresponding functions, and the author adds weight and drag to the body, making it more difficult for the robot to fly. Currently, mainstream humanoid robots often feature dexterous hands for manipulation, which requires the legs and waist to serve as a mobile platform and body support. Please provide supplementary explanations on the necessity and advantages of the configuration chosen for the robot mentioned in this paper.
2. It is mentioned in the Discussion section of Chapter 3 that the key reason for not being able to test the proposed algorithm in real flight scenarios is the lack of sensors for accurately estimating wind direction and the slow and inaccurate joint movements caused by joint delays. Has there been any attempt to perform indoor stationary hovering that does not involve the aforementioned issues?
3. According to the manuscript, the robot is powered by both fuel and batteries, with the main propulsion system using fuel and the other devices using electricity. The robot's mass is 43.3 kg (it is not specified whether this includes fuel), while the maximum thrust of the two main thrusters is only 44 kg. The two auxiliary thrusters are responsible for controlling direction and balance, each consuming up to 13 kW of power, and the robot requires a certain angle of attack for actual flight. Please conduct a detailed verification and validation of the robot's basic flight functionality to confirm its ability to fly.
4. The manuscript mentions the assumption that there is a linear relationship between energy and fuel consumption and the generated thrust, allowing the weight of the engine to be negligible. Please provide the theoretical basis for this conclusion.

Regarding contribution ② it serves as a bridge between contributions ① and ③, providing both technical and theoretical foundations for practical applications. As for contribution ③ several points require further elaboration:
1. What do the different flight configurations referred to in Figure 2e specifically entail? This should be detailed in the supplementary materials.
2. I was puzzled by Figure 3h. It states that using Lattice Boltzmann Method (LBM) simulations for two days yielded the aerodynamic area for a specific scenario with an actual error of less than 1% and that two A100 GPUs were used for the simulation. However, it also mentions an issue with computational complexity and poor scalability (the latter of which is not specifically explained in the article), rendering it unsuitable for generating large-scale aerodynamic datasets. But isn't a more accurate aerodynamic dataset preferable?
3. In the section on Deep Neural Networks for Aerodynamic Forces Prediction, it is mentioned that the Root Mean Error (RME) in the prediction validation dataset is 3x10-6. Given such high accuracy, the subsequent statement that "the validation error is still limited to below 20% of the maximum value" raises questions. What does this validation error refer to, and why is it so significant?
4. In Figure 5d, which depicts the joint error curve, how is the joint error calculated?
5. In the Robustness Analysis, could specific numerical values be provided to illustrate the differences in model accuracy between the Deep Neural Network (DNN) and the axisymmetric model? This would allow for a more quantitative assessment of the controller's tolerance to modeling errors.
6. From Figure 5d and Supplementary Figure 7d, it can be observed that the joint motion is relatively smooth during joint angle transitions. However, in the baseline controller shown in Supplementary Figures 7a and 7g, the robot experiences shaking and divergence during joint angle transitions, similar to the shaking observed in Figure 5g. What is the cause of this shaking?
7. In the physical experiments and supplementary videos, the robot's joint transitions appear smooth. Generally, there will be modeling errors when applying an axisymmetric model to a real robot, which should be consistent with its application in the DNN-based simulation environment. Why then is shaking not observed in the physical experiments but only in the simulation environment?

Version 1:

Reviewer comments:

Reviewer #2

(Remarks to the Author)

Reviewer #3

(Remarks to the Author)
Even though I don't think the answer to Comment R3.2 is very appropriate, I suggest that it be stated in the conclusion of the paper. Other revisions have improved significantly compared to the previous one.

Beyond the comment above， I have no further comments on the current version and recommend that the paper proceed to the next stage.

Open Access This Peer Review File is licensed under a Creative Commons Attribution 4.0 International License, which permits use, sharing, adaptation, distribution and reproduction in any medium or format, as long as you give appropriate credit to the original author(s) and the source, provide a link to the Creative Commons license, and indicate if changes were made.
In cases where reviewers are anonymous, credit should be given to 'Anonymous Referee' and the source.
The images or other third party material in this Peer Review File are included in the article’s Creative Commons license, unless indicated otherwise in a credit line to the material. If material is not included in the article’s Creative Commons license and your intended use is not permitted by statutory regulation or exceeds the permitted use, you will need to obtain permission directly from the copyright holder.
To view a copy of this license, visit https://creativecommons.org/licenses/by/4.0/

Dear Editor and the contributing reviewers,

We thank the editor and reviewers who contributed their time to ensure the strength of this manuscript prior to its publication. We have carefully considered each of the reviewers' recommendations and have made substantial changes to the manuscript. Further, we have included an additional experiment to validate our proposed implementation of electrostatic adhesive feathers when sliding over one another during wing morphing. We are confident that these alterations have strengthened the paper significantly. The reviewers' original comments and our direct responses (Purple) are provided below.

Additionally, the editor and reviewers may notice that the tested airflow velocities have changed from (10 m/s, 12.5 m/s, and 15 m/s) to (11.5 m/s, 14.3 m/s, and 17 m/s). During our revision process, we reanalyzed our data collection process to ensure proper data management throughout and found that we had used an old wind tunnel calibration for the flow velocities. We thus updated the reported velocities accordingly.

Thank you,

Kevin Haughn

Reviewer #2:

This paper investigates the role of electrostatic adhesion (EA) in feathered morphing wings to improve adaptive flight capabilities for small uncrewed aerial systems (UAS). The study addresses a key limitation in existing bio-inspired designs—namely, the lack of an effective mechanism to maintain feather cohesion during wing shape changes. By incorporating EA, the authors demonstrate that active feather fastening enhances maneuverability, efficiency, and stability across a range of flight speeds. However, there are also some limitations should be addressed:

While the EA system operates at low power (~3 mW), its feasibility for larger UAVs remains unclear. The adhesive forces may not scale effectively for larger wingspans, and maintaining adhesion under high aerodynamic loads could require significantly more power.

The authors appreciate the reviewer bringing to our attention an inaccuracy within our manuscript, as well as the need for considering how the EA feather design would scale

with larger wings. We have amended the presented power consumption and provided additional explanation to improve accuracy and understanding,

“The current draw was measured to be 0.3 μA at 300 V for 0.09 mW power draw for a single feather pair, or 0.012 mW/cm^2, for a total power draw of 0.72 mW for eight overlapping regions in the whole wing assembly.”

Additionally we have added lines to the Methods section considering how EA attraction will effectively scale with aircraft size and velocity, considering changes in surface area, voltage, and power consumption. [Lines: 215-216, 225-226, 285-287]

While the study discusses maneuverability improvements, it does not explore how EA-based feather fastening could be integrated into an autonomous flight control system. The impact of active adhesion on control surface deflections and overall flight stability needs further investigation.

Thank you for bringing our attention to the opportunity to discuss how our EA feathers would be implemented on a feathered morphing wing. We have added the following to the introduction to describe how control of feather morphing wings differs from traditional UAS.

[Lines: 47-50]
“These designs no longer use the aileron deflectors traditionally found on aircraft wings for roll control. Instead, the wing extension and sweep changes offered by the overlapping feather designs are used to generate roll and pitching moments. These large-scale shape changes have shown that feather inspired morphing can improve maneuverability in aerobatic UAS flight [31-33].”

Further, the implementation of EA acts to achieve greater aerodynamic performance once in a morphed state, and therefore the “active” control scheme for the EA would simply be to toggle between 300v when in a morphed state, and a low voltage or high frequency sine wave when in the process of changing morphing states to enable the feathers to slide over one another while maintaining connection. For the purposes of this work, we focused on the aerodynamic performance between the passive feathers, like those currently in use in the literature, and the fully activated EA feathers when fixed in a manner analogous to when the morphing wing would be extended, requiring greatest resistance to the aerodynamic loading. We believe that using the term “active EA” may have been misleading the reader by suggesting EA to be continuously modulated throughout flight instead of in the locking or sliding manner. We therefore have removed “active”, instead using only “EA” to represent when EA was used to fasten the feathers, and have added the following lines to the manuscript to describe how EA would be implemented within this control scheme.

Additionally we have conducted additional experimentation to validate our proposed EA control during morphing (fig. S4)

[Lines: 198-202]
“EA feather fastening can temporarily lock the wing into a desired gapless shape during flight. Alternately, shape change can be achieved by modulating the EA voltage with a high frequency waveform, reducing friction to allow the feathers to slide over one another while maintaining contact (fig. S3). This offers the adaptive benefits of wing morphing while mitigating the aerodynamic losses associated with feather gap formation.”

The supplementary material shows that some linear mixed effects (LME) models fail the normality test, and the quantification of the errors and the explanation of the reasons need to be supplemented.

The authors thank the reviewer for highlighting this discrepancy in our work. Although the size of the datasets we are working with limits the negative effects associated with non-normal residuals, following the central limit theorem, we agree that it is crucial to include uncertainty and error quantification in our modeling so readers can understand the degree of accuracy with which our results stand. Therefore, we have added significantly to the supplemental materials, uncertainty quantification through provided RMSE values and residual normality quantification through Shapiro-Wilks statistics. Additionally, we have further adjusted the models in question that previously showed non-normal residual distributions, to provide greater confidence in the statistics presented in our manuscript.

We have included another figure in the supplemental materials (fig. S2) depicting the accuracy of the linear regression models used for the pull-up maneuver simulation, and have included R^2 values and RMSE values for these models as well to inform readers of model fit and uncertainty.

Reviewer #3:

This article addresses the effect of electrostatic adhesion among the separated feather-like tailing edge structures on aerodynamic performance. The title is too ambiguous to abstract the contents of the article. It is unclear whether the suggested wing configuration can be used for a morphing wing structure or not. I also cannot fully agree with the interpretation of the measured aerodynamic data. Typically, electro-active materials require high-voltage applications for actuation, which is possible by using a heavy and large high-voltage amplifier. Due to this, their real applications are limited to demonstrations in laboratory-level research. Many papers on their applications do not mention such limitations. This article is not an exception.

The authors would like to thank the reviewer for an in-depth review of our manuscript. We have considered each of the presented concerns and adjusted the manuscript

accordingly. Revisions to the manuscript have been highlighted and are contributed to the respective reviewer comments below.

We agree that the initial title was too vague regarding the key impact of our work. Therefore, we have changed the title to “Electrostatic adhesion mitigates aerodynamic losses from feathered wing separations”.

The reviewer’s statement regarding the necessity of “heavy and large” amplifiers for the application of our electro adhesive feathers is not true. Although the amplifier used in this work was a laboratory model designed for facilitating experimentation, much smaller amplifiers (~20 g) are available for use as well. This size is appropriate for implementation on an uncrewed aerial vehicle. Further explanation and the appropriate citations are presented in our response to comment [20].

[1] Because there are some places with grammatically wrong expressions, the manuscript needs further English corrections.

The reviewer’s comment is appreciated. We have conducted a more thorough analysis to revise the English used in our writing.

[2] Many sentences include logical leaping. The authors’ own perspectives should be addressed based on the new findings in the current research, not based on the other reference papers.

We thank the reviewer for bringing to our attention the significant leaps in logic through which we attempted to lead our reader. We have made significant changes to the manuscript to improve readability and comprehension. For instance, we have more clearly outlined the structure of our experiment in the introduction [last two paragraphs] to provide readers with a greater understanding of our reasoning behind experimental decisions used to determine the source of the different performances between the EA and passive feathered wing tests.

To ensure that we are not solely relying on reference papers, we have limited many of our references to the introduction, where they are necessary to provide the reader with an understanding of the current state of the art in the field. More specifically we highlight the missing consideration for an engineered inter-feather connection mechanism.

The Results section has been significantly revised to improve clarity. This section presents only information from our own scientific findings, the two references used are simply to support that the behavior of the EA feathered wing is consistent with that of a wing with greater stiffness, as shown by our data and supported by reference 37 [Lines: 134-136]. The second reference is used to confirm that our maneuverability metric is consistent with that used in literature [Lines: 172-173].

We have rewritten the Discussion section to clarify our scientific reasoning before referring to papers that support our claims to show how our conclusions are consistent with current scientific understanding.

We have included references in our Methods section as well so that readers may be confident in the processes we used for effective and accurate experimentation and analysis.

[3] Can the current wing structure be used for morphing wings? Can it be somehow folded with the suggested way of the "feather"-like trailing edge (TE) assembly, which is shown in Figure 1(B) and Fig. S1?

Thank you for highlighting the need for further clarity in our experimental design. Your question has illuminated that we hadn't sufficiently explained how our work relates to, and may be implemented in, a morphing wing design. The leading edge of the tested wing structure is rigid, and therefore unable to change shape or fold like the reviewer has suggested. Instead, our work is meant to investigate the impact of a feather fastening mechanism (here EA) on a wing while it is fully extended and thus experiencing the greatest aerodynamic loading (rather than in a folded wing when the feathers overlap one another more completely). We have added to the following sentences to highlight foldable feather morphing wings that exist in the literature, and describe how our EA feather design could be integrated into those systems. In the introduction we describe the current state of the art in feather morphing wings. [Lines: 198-202] in the Discussion section describes how modulating voltage for EA can be used to maintain connection while changing shape, and [Lines: 310-318] in the Methods subsection "*Shear traction testing*" describe the additional testing we performed to support the feasibility of using such a method. This is further supported by a new figure (fig S3) in the supplemental material.

[4] Birds actively flap and rotate their wings for various modes of flight, where bending and torsional stiffnesses are provided by muscles and bones in the inboard wing. A typical fixed wing also requires both bending and torsional stiffnesses to endure large bending and torsional loadings. Therefore, a closed wing cross section is preferred in a fixed wing, something like the baseline wing in Figure 2A. In the current feathered wings, how was the torsional load supported in the experiments?

We thank the reviewer for this important consideration for structural design of our wing. For the tested flow velocities, we observed no torsional deflection for any of the tested wings. The bending deflection was minimal (< 5 degrees) and comparable for each wing, and was likely due to the stiffness of the ¼-20 aluminum mounting rod rather than the geometry or material of the wings themselves. This small and similar deflection was not enough to cause significant inaccuracies in the data or detract from comparing performance between wings. The following lines were added to address the reviewers concern [Lines: 354-357]

“During testing no torsional deflection was observed for any of the tested wings, and bending deflection was minimal (< 5 degrees) and consistent across tested wings. Since only longitudinal aerodynamic forces and moments were considered, this small deflection was considered acceptable.”

[5] It seems obvious that the feathered wing with electrostatic adhesion (EA) aerodynamically performs better because the fluctuation of TE can be smaller with the adhesion. Thus, the aim of the work should be clear. Is it to see how much the EA contributes to the improvement of aerodynamic performance compared to the case with no EA. Or does it aim to show that the feathered wing with EA outperforms the baseline wing?

Thank you for bringing this source of confusion to our attention. Since rigid engineered airfoils have been thoroughly optimized to improve traditional flight characteristics, we cannot expect unoptimized feathered wings to outperform these wings in traditional aerodynamic metrics. However, unlike the rigid baseline wings, feathered wings have the ability to change shape for greater adaptability, and even maneuverability, in changing mission spaces or environments. Until now, the field has largely ignored the formation of gaps that occurs between the engineered feathers used in these morphing wings. Therefore, we aimed to develop and investigate a method to actively fasten these feathers to quantify the importance of eliminating separation between feathers to reduce the loss of maneuverability and other aerodynamic properties. We incorporated the two baseline wings (one from a previous engineered design, and another we developed to share the same airfoil profile as the feathered wings) so that we could determine whether to contribute changing aerodynamic qualities to the new airfoil geometry, TE stiffness, or airflow between feathers due to gap formation.

To make this clearer to our readers, we have de-emphasized comparisons to the baseline wings and re-emphasized the comparisons between feathered wings. Additionally, significant rewriting of the introduction and results section now emphasizes the purpose of the two rigid baseline wings to decipher the contributions of compliance and geometry to the aerodynamic effects, and to serve as baselines from which to compare the effect of EA on the feathered wing. [Lines: 68-78, 84-87, 100-109, 116-126, 187-196]

[6] It seems natural that the CL max of the feathered wing with EA (300V) is larger than those of the other feathered wing. Even in this case, it may be plausible that the authors include the possible reasons more rigorously. Still, the max CL of the feathered wing with EA (300V) is lower than that of the baseline wing for all air speeds. The slightly large CL slope of the feathered wing with EA (300V) or other data do not properly support that the feathered wing performs better than the baseline wing.

Thank you for bringing to our attention the unclear motivation for our paper. It is known that streamwise gap formation leads to reduced lift in rigid wings, and yet mitigating this occurrence in feather-based morphing wing designs has not yet been addressed through engineering means. The purpose of this paper is to show the aerodynamic

benefits of using EA to prevent gap formation on a feathered wing, thus highlighting the importance of considering gap mitigation when designing morphing aircraft.

We have added the following to the introduction to clarify this point [Lines 53-57]: "Streamwise gap formation on a rigid wing can reduce lift as angle of attack, α, increases [34-36]. Similarly, disconnecting thin feather-like structures reduces their combined stiffness, which can result in a more compliant trailing edge and ultimately reduce lift at higher flow speeds [37]. Although these effects can benefit gust alleviation, the result limits an aircraft's ability to rapidly change direction. Therefore, optimal performance during aerobatic flight requires feather connection to be maintained."

Similarly to [5], we expect that the reviewer's comment regarding the baseline wing was a result of our failure to clearly describe the role of the two rigid baseline wings. We have added substantial clarification of this point in the manuscript.

[7] When 300V is applied, the electro-active material may deform. If the material layers are not placed in the neutral surface in the thickness direction, the deformation may create bending of the TE. Was this observed in the measurements?

We thank the reviewer for brining this concern to our attention. The combined acting distance of the electroadhesive with a voltage of 300 V was insufficient to overcome the feather stiffness and cause significant bending deflection. We have added the following line to the Methods section [Lines: 279-281]. "This field strength was sufficient for adhesive purposes, but was not strong enough to produce significant deformation and trailing edge feather deflection due to EA activation was not observed during testing."

[8] Because CL was used in figure 2, the statement in lines #80-82 should be modified such that the explanation is based on CL. The same for line #82. If the comparison is kept in terms of lift, then the data of lift should be compared in figure 2. With the current data presentation, I cannot be convinced with the current explanation.

Thank you for bringing this inconsistency to our attention. The verbiage has been changed in the appropriate sentences to use "C_L " instead of "lift" to better match the data presented in Figure 2.

[9] The comparison of the rate of CL_max change due to the speed increase is not so important because the CL_max of the active feathered wing was much lower than those of the baseline wing at low speeds.

Thank you for bringing this concern to our attention. This suggestion follows the concerns raised before in [5] and [6]. The primary goal of this work is to show the aerodynamic effect offered by EA for a feathered wing, and thus is compared to the passive feathered wing. We provide the baseline wing for comparison to show that the EA wing generates a similar positive $C_{L\text{-}max}$ rate of change behavior, rather than the

negative rate of change produced by the passive feathered wing. Presenting this preferred behavior generated by incorporating EA on the feathered wing is important as it directly supports the motivation for this work stated in the introduction and abstract.

Additional clarification on this comparison has been included in the abstract.

[10] The statements in lines #105-107 seem obvious. Still, it is better to explain the science behind it.

We thank the reviewer for suggesting we provide greater scientific explanation to support this statement. We have significantly rewritten the subsection within the Results titled *Aerodynamic characteristics vary with velocity* to clarify the primary findings of this section so that the statement of interest is more clearly supported for the reader. [Lines 133-136]

[11] Similar to my comment in [9], the L/D of the baseline wing is always higher than those of the feathered wings. I cannot understand the statement in line #133-134. Explain with scientific proofs.

Similar to previous concerns regarding the comparison to the baseline, the primary motivation of this manuscript is to improve on the passive feather design by incorporating EA feather fastening.

An additional interesting piece with respect to L/D is how the efficiency of the two rigid wings decreased as velocity increased, whereas the more compliant feathered wings improved in efficiency as the velocity increased. Notably, EA provided an additional boost on that efficiency gain to achieve 95% of the efficiency achieved by the engineered airfoil geometry. This is impressive since feathered wings are designed for the ability to change shape, and not necessarily optimized for efficiency as traditional engineered airfoils are. Additionally, the performance of the rigid plate wing (which shares airfoil geometry of the feathered wing) provided a comparison to show that structural compliance is a key part of improving efficiency above the limits of the airfoil geometry itself.

In addition to the previous adaptations made to the manuscript to highlight the motivation for our comparisons to the baseline, we have rewritten the appropriate paragraph in the results section [Lines: 146-157] to more clearly convey this to the reader.

[12] How can the static stability of an airplane be explained with only Cm? As defined here, how is a wing statically stable when Cm decreases as AOA increases? Cm is typically negative as shown in Figure 3(D). Therefore, for an increase in AOA of a wing, the wing tends to go back to the original AOA. With this, we may define that the wing is statically stable with such Cm characteristics. For the active EA, the wing tends to have

a larger magnitude of Cm than the other feathered wings. However, the magnitudes of the max Cm of the feathered wing are smaller than those of the baseline wing for AOAs less than about 12 degrees. For AOAs larger than about 12 degrees, there are lots of fluctuations of data. Thus, we cannot trust the data. Their slope cannot be a measu.re of static stability.

Thank you for bringing this to our attention. Although the static stability of an aircraft is directly related to the slope of the C_m vs Angle of attack curve, your comment brought to our attention that presenting this data as a consideration for EA's impact on aircraft stability through manipulating the wing's contribution to the total aircraft pitching moment is not sufficient. The wing's contribution to the aircraft pitching moment, and therefore how the horizontal stabilizer must respond to this contribution, is contingent on many design variables including the location of the center of gravity, the distance to the aircraft tail, and the general design of the aircraft's tail (including size and incidence angle). Considering each of these potential design variables would greatly shift the significance of any findings determined through wing pitching moment alone and therefore diminish any comparison between the activated EA and passive feathered wing. For this reason, after considerable deliberation, we have decided that EA's contribution to aircraft stability cannot be effectively considered within the scope of this manuscript and therefore any discussion related to the topic has been removed.

[13] I assume that the results on maneuverability are based on simulation. The explanation and the assumptions in lines #163 to 169 are unclear to me. Is it for a fixed-wing A/C? Were the measured CL, CD, and Cm data implemented for the simulation? Again, the authors should address the science behind figure 4 to strengthen the article.

We thank the reviewer for bringing to our attention to our lack of clarity when presenting the simulation findings. We have clarified the description of our kinematics model and the relevant assumptions [Lines: 160-168, 175-178].

"Enforcing electrostatic connection between feathers generated significant aerodynamic improvements for the feathered wing that can contribute to greater maneuverability in flight. To illustrate this effect, we constructed two longitudinal point-mass dynamics flight models to simulate rapid pull up flight trajectories for the feathered wing with and without EA. These models assumed the wing was the primary source of lift and drag, neglecting the aerodynamic characteristics of a fuselage and tail. We used linear regression to model the C_L and C_D measurements and predict the lift and drag generated throughout the linear flight regime (-5° ≤ α ≤ 12° ; 11.5 m/s ≤ U ≤ 17 m/s) (fig. S2). Additionally, the model assumed effective pitch control was achieved by an external control surface, such as an elevator located on the aircraft's tail."

"We used PID feedback control to effectively adjust aircraft pitch rate so that angles of attack would consistently approach and then maintain an incidence of 10° during the pull up maneuver. This allowed the simulated aircraft to achieve high lift while remaining within the linear flight regime, thus preventing the occurrence of stall and maintaining model accuracy."

A more in-depth description of how we developed the simulation is provided in the Methods subsection titled *Maneuver Simulation.*

[14] I cannot find any figure for the peeling test of the EA materials. Include more explanations on it. More specific information on the load cell information (line #295 and 302) should be included, such as manufacturer, max capacity, and resolution.

Thank you for bringing this to our attention. We agree that including a figure to describe our shear traction experiment would be beneficial for the reader. We have thus included an additional figure in the supplemental material (Fig. S3A). Additionally we have added further description of the shear testing in the Methods section [Line: 300], including load cell information.

[15] Was the load cell rotated along with the wing. In this case, the load cell axis is rotated too, which causes wrong interpretations of the load cell signals. Include more details on the installation.

Thank you for mentioning this concern. The load cell was rotated along with the wing during experimentation and the appropriate rotation matrices were used to determine the lift and drag of the wing with respect to the angle of attack. We have added the following statement to clarify this in the manuscript.

[Lines 334-336]: "After data collection, the load cell measurements associated with the x and y axes were rotated by the appropriate angles of attack to ensure proper lift and drag measurements were recorded."

[16] For wind tunnel experiments, we typically install a wing in the middle of test section. In this experiment, the wings were installed on the floor of the test section, where the inflow speed might not be uniform over the wingspan dur to the friction on the floor. Was it considered in the experiments?

Thank you for bringing this concern to our attention. Half-span wind tunnel testing is often performed with the model mounted directly to the wall of the wind tunnel. However, this can lead to boundary layer effects from the wind tunnel wall. It is for this reason, we mounted our half-span wing approximately 4 inches from the wall (or ceiling in our case) to ensure the wing was outside of boundary layer effects. The following sentence was included to improve clarity.

[Lines: 324-326] "The wing root and base plate were mounted approximately four inches from the test section ceiling to ensure the entire wing remained outside of the wind tunnel boundary layer during testing."

[17] Include references for equations (4)-(9). I cannot understand how these equations came out. Can they (equations 4 and 5) be called a linear regression model even though alpha squared and U squared terms are in there?

Thank you for raising this concern regarding our equations used to model the flight trajectories. We agree that it is important that our readers understand the nature of these formulas.

Firstly, equations 4 and 5 are derived from the traditional linear equation used for linear regression,

$$Y = A_0 + A_i X_i,$$

where Y is the dependent variable, A_0 is the intercept, and A_i is the array of i coefficients (or slopes) for each respective dependent variable X_i. It is still considered linear regression, because it solves for the A values (intercept and slopes) in this linear relationship regardless of the form of the dependent variables X_i. This includes polynomials and interactions between variables, allowing linear regression to be useful for modeling more complex functions. I have added the following citation for the Ordinary Least Squares linear regression used here.

Galton, Francis. "Regression towards mediocrity in hereditary stature." *The Journal of the Anthropological Institute of Great Britain and Ireland* 15 (1886): 246-263.

We added the following lines to further describe these linear regression models.

[Lines: 367-368] "where A_n and B_n are the nth coefficient of each model and $n = 0$ represents the intercept of each model. Angle of attack, α, and flow velocity, U, were the basis of each independent variable."

Regarding equations 6 – 9, we have rewritten the paragraph pertaining to the point-mass flight dynamics model, and added intermediate equations to provide a more descriptive account of the flight simulation. We also supplied the appropriate reference from which these equations were derived [Lines: 372-390].

Pamadi, B. N. (2004). *Performance, stability, dynamics, and control of airplanes*. Aiaa.

[18] For creating a level flight, the pitching moment equilibrium should be also satisfied. For longitudinal maneuvering, pitching motion should be considered. Equation (10) is for force equilibrium in the flight direction. Is it good enough to describe the pitching maneuvering flight? Are T and Tt different?

Thank you for bringing to our attention this potential source of confusion for our readers. It is true that when modeling the flight of full aircraft, the pitching moment should be considered. However, as described in our response to number [13], our flight simulation is built from a simple 2D point-mass model, which is appropriate for modeling flight using wing data alone. Since we wanted to focus on the impact of EA on the feathered

wing, we did not want to limit our results to one theoretical aircraft design. Pitch control is ultimately determined through the design of the tail and center of gravity for the full aircraft and thus would vary significantly between designs. Therefore, we decided to assume effective pitch control would be achieved for each simulated flight to provide an equivalent basis of comparison for each wing.

Since the aircraft remains within the linear flight regime, where angle of attack is less than stall (α < 12), angle of attack change rate, $\dot{\alpha}$, was small, this model is appropriate for describing the pitching maneuver.

We have added significantly to the methods subsection *Maneuver Simulation* to emphasize these assumptions. [Lines: 391-394, 407-409] .

[19] Equations (11) should be properly explained by calling out references. The same for the other equations.

Thank you for highlighting this potential source of confusion for our readers. We have included the following reference for the PID feedback equation.

Franklin, G. F., Powell, J. D., Emami-Naeini, A., & Powell, J. D. (2002). *Feedback control of dynamic systems* (Vol. 4). Upper Saddle River: Prentice hall.

[20] Actuation of EA materials require a high-voltage amplifier as in this work, which requires a heavy high-voltage amplifier. Therefore, the drawbacks of its use in a morphing wing should be addressed.

We appreciate the reviewers comment here, as readers may conclude that the large laboratory amplifier used in our experimentation are the only means of achieving the electrostatic adhesion necessary for our feathered wing design, thus making its use for small scale aircraft impossible. We have therefore included two additional references to ensure our readers that this is not the case,

S. K. Mitchell, T. Martin, C. Keplinger, A pocket-sized ten-channel high voltage power supply for soft electrostatic actuators. Adv. Mater. Tech. 7, 2101469 (2022).
Developed a 10-channel, smartphone-sized high voltage power supply capable of producing up to 10 kV.

Stephan-Daniel Gravert *et al.* Low-voltage electrohydraulic actuators for untethered robotics. Sci. Adv. 10, eadi9319 (2024). DOI:10.1126/sciadv.adi9319
Developed a two-channel 900 V high voltage power supply 42 mm x 19 mm x 22 mm in size or 11 cm^3 in volume and weighing 15.5 g

Additionally, we have included lines in the Methods discussing this concern [Lines: 287-290] and a supplementary table that includes the mass of each component needed to produce the required voltage for EA (Table S1).

These materials were pulled from an existing small fixed wing UAV we built for another flight tested EA application. Although this application will not be mentioned in the manuscript since it has not been approved for public release, we mention it here assure the reviewer and editor that mass is not a barrier for EA application on small aircraft.

[21] The titles of references [5], [27],[32],[39] [42], and [46] are missing. Please carefully check the others too.

Thank you for bringing this mistake to our attention. We have carefully looked through the reference section to ensure all references are appropriately written.

Reviewer #4:

The article proposes research on an electrostatic adhesion-based variable trailing-edge wing that can control the trailing-edge gap through voltage changes, thereby affecting the
aerodynamic characteristics of the wing. The topic, as well as the research itself, is interesting. However, there are certain shortcomings in the manuscript's content.

Firstly, in the section titled "Feathered Wings Provide Efficiency and Stability," the concept of static stability in aircraft design is usually determined by the relationship between the aircraft's aerodynamic center and its center of gravity. It is typically not considered for an isolated wing. Since the manuscript does not specify the aircraft's center of gravity, I believe the moment analyzed in this paper refers to the moment of the aerodynamic center relative to a fixed chordwise position of the sensor. This moment is influenced by the shift of the aerodynamic center due to the passive deformation of the trailing edge, but this does not indicate that the feathered wing has better static stability. Even if stability issues are considered, the method of generating a stabilizing moment through active control of trailing-edge feather deformation to create aerodynamic changes should not be defined as static stability.

Thank you for your insightful concerns regarding our manuscript. Initially we believed that discussing the wing's contribution to the pitching moment, and therefore static stability, of an aircraft would provide some interesting insight regarding the geometry and compliance of our feathered wing. However, you brought up a good point that the aircraft's static stability will ultimately depend on the rest of the design. Therefore, since our goal is to focus on the effect of using EA for feather connection on a feathered wing, we agree that commenting on aircraft stability falls outside the scope of this manuscript and have therefore removed the appropriate section.

Secondly, although the article analyzes the differences between the active variable trailing edge wing, passive variable trailing-edge wing, and rigid wing models, it does

not provide the control logic for the active wing. What is the control logic for the electrostatic adhesion
feathered wing under conditions such as variations in incoming flow and gusts, and what kind of deformation does it produce? The author should clearly specify these prerequisites for different operating conditions to improve the reproducibility of the work.

Thank you for your comment. We now realize that our terminology, specifically the use of “active EA” was misleading to our readers as it suggests that there is a form of active control in use during our experimentation. All measurements were conducted at constant voltages (150V, 200V, and 300V) and 0V for the passive case. After determining that the intermediate voltages produced gradual degradation of inter-feather connection as angle of attack increased Fig 2D, the remainder of comparisons focused on the difference between the passive feathers 0V and fully activated EA feathers (300V). We make these comparisons to emphasize the importance of incorporating some form of feather connection for feathered morphing wings which currently do not prevent gap formation from occurring. The ‘active’ component of our EA feathers is the ability to modulate the voltage so that movement between the feathers would enable wing shape change.

To clarify this, we have rewritten much of the manuscript to emphasize the primary goal of this work and removed the term ‘active EA’ to prevent misleading our readers. Additionally, we have included the following lines to describe a potential control logic for implementing EA on a morphing wing, and have completed additional experimentation to validate the morphing concept (fig S3).

[Lines: 198-202]
“EA feather fastening can temporarily lock the wing into a desired gapless shape during flight. Alternately, shape change can be achieved by modulating the EA voltage with a high frequency waveform, reducing friction to allow the feathers to slide over one another while maintaining contact (fig. S3). This offers the adaptive benefits of wing morphing while mitigating the aerodynamic losses associated with feather gap formation.”

Thirdly, the article only compares the lift-to-drag ratio (L/D) and moment characteristics from an engineering perspective. It is recommended to delve deeper into the underlying mechanisms from the aspects of aerodynamics and control. The current comparison in the
article does not exclude the possibility that the findings are only valid for the specific wing
size used in the study.

Thank you for your suggestion. Streamwise gap formation in a rigid wing [Sigrest; Ref 34 and 35], as well as the impact of feather like compliance [Gamble; Ref 37], have been scientifically explored. Sigrest investigated, through computational fluid dynamics and experimentation, the aerodynamic characteristics of varying streamwise gaps in a

rigid wing as a means for rapid descent and possible roll control at high angles of attack. They found the expected reduced aerodynamic performance within typical flight regimes. Gamble found that a compliant trailing edge (informed by passive shape change of feathers under loading) experienced reduced aerodynamic loads due to passive trailing edge deflection as Reynolds number (in this case varied by velocity) increased.

These scientific findings were the core influence for our investigation to find an engineering solution to gap formation in feathered wing designs, which are currently at the forefront of small morphing uncrewed aerial vehicle design (investigated at Reynolds numbers between 10^5 and 1.5×10^5). It was from this engineering perspective that we chose *Communications Engineering* as the journal for publication.

We have thus included a more direct reference to these prior scientific findings to provide greater confidence to the reader that the findings presented here are of a broader scientific basis, and therefore applicable beyond the wing used in our study.

[Lines: 53-57]
"Streamwise gap formation on a rigid wing can reduce lift as angle of attack, α, increases [34-36]. Similarly, disconnecting thin feather-like structures reduces their combined stiffness, which can result in a more compliant trailing edge and ultimately reduce lift at higher flow speeds [37]. Although these effects can benefit gust alleviation, the result limits an aircraft's ability to rapidly change direction. Therefore, optimal performance during aerobatic flight requires feather connection to be maintained."

Additionally, regarding the scale of this work, we selected the size and flow velocities to ensure our measurements were conducted at a range covering the aerodynamic scale (measured by Reynolds number) of the current state of the art. We have added the following lines to clarify our reasoning behind the scale of this work.

[Lines: 44-47]
"Recently, there has been a push toward greater biological inspiration in UAS design, flying at lower Reynolds numbers (Re) and using overlapping feathers, or feather-like plates, to achieve relatively large-scale shape changes without sacrificing structural integrity [25-31]."

[Lines: 79-82]
"Aerodynamic force measurements were collected on each half-span wing model in a wind tunnel at angles of attack ranging from -5° to 20°, and at a range of flow velocities (11.5 m/s, 14.3 m/s, and 17 m/s) ensuring comparable aerodynamic scale to the current state of the art (*30, 31*) ($10^5 \lesssim Re \lesssim 1.5 \times 10^5$)"

Finally, we have added a paragraph to the Methods to describe how EA connection will naturally scale as the size of the wing increases, and how voltage can be scaled to adjust for increased aerodynamic loads associated with velocity.

[Lines: 291-298]
“Although experimentation was limited to the scale of the wing described, equations 1-4 provide insight regarding the relationship between aerodynamic loading and EA strength as scale changes. Aerodynamic loading and EA attraction force increase linearly with surface area, suggesting the findings presented in this work would effectively scale with changes in wing and feather size. Additionally, power consumption will scale linearly with surface area according to 0.012 mW/cm2. Aerodynamic loads scale quadratically with flow velocity and EA attraction forces scale similarly with voltage. This suggests that wing designs developed for flight at higher velocities can account for the increased loads by applying greater voltages to the EA system.”

Dear Dr. Rosamund Daw,

Thank you for considering our manuscript for publication in Communications Engineering. We were glad to see that the reviewers were satisfied with our revisions. We have made a few formatting adjustments in accordance with the editorial checklist, added scale bars to the suggested figures, and included an additional supplemental figure (fig. S10) providing box-and-whisker plots depicting the raw data distributions.

Thank You,

Kevin Haughn

nature portfolio

Peer Review File

Electrostatic adhesion mitigates aerodynamic losses from gap formations in feathered wings

Corresponding Author: Dr Kevin Haughn

Version 0:

Reviewer comments:

Reviewer #2

(Remarks to the Author)
This paper investigates the role of electrostatic adhesion (EA) in feathered morphing wings to improve adaptive flight capabilities for small uncrewed aerial systems (UAS). The study addresses a key limitation in existing bio-inspired designs —namely, the lack of an effective mechanism to maintain feather cohesion during wing shape changes. By incorporating EA, the authors demonstrate that active feather fastening enhances maneuverability, efficiency, and stability across a range of flight speeds.

However, there are also some limitations should be addressed:

While the EA system operates at low power (~3 mW), its feasibility for larger UAVs remains unclear. The adhesive forces may not scale effectively for larger wingspans, and maintaining adhesion under high aerodynamic loads could require significantly more power.

While the study discusses maneuverability improvements, it does not explore how EA-based feather fastening could be integrated into an autonomous flight control system. The impact of active adhesion on control surface deflections and overall flight stability needs further investigation.

The supplementary material shows that some linear mixed effects (LME) models fail the normality test, and the quantification of the errors and the explanation of the reasons need to be supplemented.

Reviewer #3

(Remarks to the Author)
Please read the attached file.

Reviewer #4

(Remarks to the Author)
The article proposes research on an electrostatic adhesion-based variable trailing-edge wing that can control the trailing-edge gap through voltage changes, thereby affecting the aerodynamic characteristics of the wing. The topic, as well as the research itself, is interesting. However, there are certain shortcomings in the manuscript's content.

Firstly, in the section titled "Feathered Wings Provide Efficiency and Stability," the concept of static stability in aircraft design is usually determined by the relationship between the aircraft's aerodynamic center and its center of gravity. It is typically not considered for an isolated wing. Since the manuscript does not specify the aircraft's center of gravity, I believe the moment analyzed in this paper refers to the moment of the aerodynamic center relative to a fixed chordwise position of the sensor. This moment is influenced by the shift of the aerodynamic center due to the passive deformation of the trailing edge, but this does not indicate that the feathered wing has better static stability. Even if stability issues are considered, the method of generating a stabilizing moment through active control of trailing-edge feather deformation to create aerodynamic changes should not be defined as static stability.

Secondly, although the article analyzes the differences between the active variable trailing-edge wing, passive variable trailing-edge wing, and rigid wing models, it does not provide the control logic for the active wing. What is the control logic for the electrostatic adhesion feathered wing under conditions such as variations in incoming flow and gusts, and what kind of deformation does it produce? The author should clearly specify these prerequisites for different operating conditions to

improve the reproducibility of the work.

Thirdly, the article only compares the lift-to-drag ratio (L/D) and moment characteristics from an engineering perspective. It is recommended to delve deeper into the underlying mechanisms from the aspects of aerodynamics and control. The current comparison in the article does not exclude the possibility that the findings are only valid for the specific wing size used in the study.

Version 1:

Reviewer comments:

Reviewer #2

(Remarks to the Author)
Thanks for revising the manuscript.

Reviewer #4

(Remarks to the Author)

Open Access This Peer Review File is licensed under a Creative Commons Attribution 4.0 International License, which permits use, sharing, adaptation, distribution and reproduction in any medium or format, as long as you give appropriate credit to the original author(s) and the source, provide a link to the Creative Commons license, and indicate if changes were made.
In cases where reviewers are anonymous, credit should be given to 'Anonymous Referee' and the source.
The images or other third party material in this Peer Review File are included in the article’s Creative Commons license, unless indicated otherwise in a credit line to the material. If material is not included in the article’s Creative Commons license and your intended use is not permitted by statutory regulation or exceeds the permitted use, you will need to obtain permission directly from the copyright holder.
To view a copy of this license, visit https://creativecommons.org/licenses/by/4.0/
